# Effects of the duration of postresuscitation hyperoxic ventilation on neurological outcome and survival in an asphyxial cardiac arrest rat model

**DOI:** 10.1038/s41598-019-52477-y

**Published:** 2019-11-11

**Authors:** Tongyi Hu, Jianjie Wang, Shuangwei Wang, Jingru Li, Bihua Chen, Feng Zuo, Lei Zhang, Yuanyuan Huang, Yongqin Li

**Affiliations:** 1 Department of Biomedical Engineering and Imaging Medicine, Army Medical University, Chongqing, 400038 China; 2 Shenzhen Dashen Institute of Biomedical Engineering Translation, Shenzhen, 518060 China; 3 Department of Information Technology, Southwest Hospital, Army Medical University, Chongqing, 400038 China; 4 Department of Emergency, Southwest Hospital, Army Medical University, Chongqing, 400038 China; 5 Department of Neurology, Southwest Hospital, Army Medical University, Chongqing, 400038 China

**Keywords:** Hypoxic-ischaemic encephalopathy, Stroke

## Abstract

Cardiac arrest leads to sudden cessation of oxygen supply and cerebral hypoxia occurs when there is not sufficient oxygen supplied to the brain. Current Guidelines for adult cardiopulmonary resuscitation (CPR) and emergency cardiovascular care recommend the use of 100% oxygen during resuscitative efforts to maximize the probability of achieving the return of spontaneous circulation (ROSC). However, the optimal strategy for oxygen management after ROSC is still debatable. The aim of the present study was to evaluate the effects of the duration of post-resuscitation hyperoxic ventilation on neurological outcomes in asphyxial cardiac arrest rats treated with targeted temperature management (TTM). Asphyxia was induced by blocking the endotracheal tube in 80 adult male Sprague-Dawley rats. CPR begun after 7 min of untreated cardiac arrest. Animals were randomized to either the normoxic control under normothermia (NNC) group or to one of the 4 experimental groups (n = 16 each) immediately after ROSC: ventilated with 100% oxygen for 0 (O_2__0h), 1 (O_2__1h), 3 (O_2__3h), or 5 (O_2__5h) h and ventilated with room air thereafter under TTM. Physiological variables were recorded at baseline and during the 6 h postresuscitation monitoring period. Animals were closely observed for 96 h to assess neurologic recovery and survival. There were no significant differences in baseline measurements between groups, and all animals were successfully resuscitated. There were significant interactions between the duration of 100% oxygen administration and hemodynamics as well as, myocardial and cerebral injuries. Among all the durations of hyperoxic ventilation investigated, significantly lower neurological deficit scores and higher survival rates were observed in the O_2__3h group than in the NNC group. In conclusion, postresuscitation hyperoxic ventilation leads to improved PaO_2_, PaCO_2_, hemodynamic, myocardial and cerebral recovery in asphyxial cardiac arrest rats treated with TTM. However, the beneficial effects of high concentration-oxygen are duration dependent and ventilation with 100% oxygen during induced hypothermia contributes to improved neurological recovery and survival after 96 h.

## Introduction

Oxygen plays a pivotal role in medicine, and supplemental oxygen therapy remains central to care in many life-threatening neurological emergency situations. Cardiac arrest, which is the sudden cessation of spontaneous ventilation and circulation, represents the most urgent need for rapid oxygen delivery to organs^1^. Since adequate oxygen supply is necessary to restore the energy state of the heart and to maintain the energy state of the brain in order to minimize ischemic and hypoxic damage, the latest Guidelines for adult cardiopulmonary resuscitation (CPR) and emergency cardiovascular care therefore emphasize proper oxygen administration and blood flow maintenance. Because blood flow is the main factor affecting oxygen delivery, it is important to maximize oxygen content in the arterial blood by maximizing inhaled oxygen concentration during resuscitative efforts^2,3^.

After the return of spontaneous circulation (ROSC) is achieved, the Guidelines advocate using the maximal available oxygen concentration until resources are available to monitor oxyhemoglobin saturation and to titrate FiO_2_ to avoid hypoxia^4,5^. In clinical practice, patients may receive 100% oxygen gas during the early postresuscitation period until they are transported to the intensive care unit and mechanically ventilated. This period may vary from thirty minutes to several hours^6,7^.

Recently, concerns about the potentially harmful effects of hyperoxia have been expressed^8^. Although animal studies have demonstrated that the administration of high concentrations of oxygen might increase postresuscitation brain damage, clinical studies of cardiac arrest have reported controversial results^9–11^.

In consideration of the restricted and conflicting data reported from human studies, the current animal study was aimed to assess the effects of the duration of hyperoxic ventilation on outcomes in a cardiac arrest rat model of asphyxia under targeted temperature management (TTM). TTM was used because it is the current standard therapy for asphyxiated neonates with hypoxic-ischemic encephalopathy, and TTM is recommended by the Guidelines for all comatose adults resuscitated from cardiac arrest^2,12,13^. We hypothesized that neurological recovery and survival are greatly affected by the duration of postresuscitation high concentrations of oxygen administrated to animals treated with hypothermia.

## Methods

This prospective, randomized observational animal study was approved by the Laboratory Animal Welfare and Ethics Committee of the Army Medical University. Eighty healthy adult male Sprague-Dawley rats weighing between 282 and 365 g were used for this study. The primary aim of the study was to determine whether the duration of postresuscitation hyperoxia affects neurological recovery and mortality rate in animals treated with hypothermia. The secondary aim was to identify the optimal duration of hyperoxic ventilation beginning immediately after resuscitation. All animal experiments were performed in strict accordance with guidelines from the Regulations for the Administration of Affairs Concerning Experimental Animals and efforts were made to minimize the suffering of the animals.

### Animal preparation

The animals were housed in a conventional facility under controlled temperature and humidity conditions with a 12 h normal dark-light cycle with free access to water and chow. All animals were fasted overnight with the exception of free access to water. Anesthesia was initiated with an intraperitoneal injection of pentobarbital (45 mg/kg). Additional doses of 10 mg/kg were administered at intervals of 1 h or when required to maintain anesthesia. After placing the animals on a surgical board in the supine position, the tracheas were intubated through a tracheotomy with a 14-gauge cannula, and the animals were mechanically ventilated with a tidal volume of 0.65 ml/100 g at an FiO_2_ of 0.21 (ALC-V8, Alcott Biotech Co. Ltd, Shanghai, China). A PE-50 catheter was advanced into the right femoral artery for measurement of arterial pressure and blood sampling. The left femoral vein was also cannulated with an additional PE-50 catheter to allow for the administration of fluids and drugs. All catheters were flushed intermittently with saline solution containing 2.5 IU/ml heparin. Three subcutaneous needle electrodes were inserted into the limbs to measure electrocardiogram (ECG). Four subdermal needles were inserted into the left and right surfaces of the skull to record the electroencephalogram (EEG) signals^14^.

### Experimental procedures

After the collection of baseline data, pipecuronium (0.1 mg/kg i.v.) was administered to induce respiratory paralysis. The mechanical ventilator was disconnected, and the endotracheal tube was clamped to induce asphyxia. Cardiac arrest was defined as a mean arterial pressure (MAP) decrease to less than 30 mmHg with either pulseless electrical activity or asystolic rhythm, occurring approximately 3 min after asphyxia^15^.

CPR, including chest compression and ventilation, was initiated after 7 min of untreated cardiac arrest. Manual external chest compressions were performed by the same investigator at a rate of 240 compressions/min with a depth of 25–30% of the anterior posterior diameter of the animal’s chest. Coincident with the start of the precordial compressions, the animals were mechanically ventilated at a frequency of 80/min with a tidal volume of 0.6 ml/100 g and an FiO_2_ of 1.0. A dose of epinephrine (0.02 mg/kg) was injected 30 s after the start of CPR. Defibrillation was attempted with a single 2-J rectilinear biphasic shock (M-Series, Zoll Medical corporation, Chelmsford, MA, USA) if the cardiac rhythm was shockable after 2 min of CPR. Resuscitation efforts were continued until the spontaneous pulse was observed with arterial tracing and when the MAP reached above 60 mmHg for at least 5 min, or the animal was pronounced dead after a total of 10 min of CPR.

The animals were randomized to either the normoxic control under normothermia (NNC) group or one of the 4 experimental groups immediately after ROSC and were monitored in an intensive care setting for 6 h (n = 16 each): ventilated with 100% oxygen for 0 (O_2__0h), 1 (O_2__1h), 3 (O_2__3h), or 5 (O_2__5h) h and ventilated with room air thereafter. The animals in the NNC group were ventilated with room air, with the core temperature maintained at 37.5 ± 0.3 °C. For the animals assigned to the experimental groups, surface cooling was initiated immediately after ROSC with the aid of ice packs and with an electrical fan for TTM. Once the target temperature reached 33.0 °C, it was maintained over the first 2 h of postresuscitation and then gradually returned to 37.5 °C over a rewarming period of 2.5 h^16^.

All catheters, including the endotracheal tube, were removed, and the wounds were surgically sutured 6 h after resuscitation. The animals were then returned to their cages and observed for 96 h.

### Measurements

Arterial pressure and lead II ECG were continuously measured by a multiparameter monitor (Model 90369, Spacelabs, Snoqualmie, WA, USA). EEG signals were amplified and conditioned by a two-channel EEG differential preamplifier (PRE-ISO.EEG100, Xiangyun Computing Technology, Beijing, China). The arterial pressure and, ECG and EEG waveforms were synchronously recorded with a PC-based data acquisition system (DATAQ Instruments Inc., Akron, OH, USA). The characteristics of the earlier postresuscitation EEG, including the onset time of the EEG burst (OTOB), time to normal EEG trace (TTNT), and information quantity (IQ), were quantitatively analyzed^12^. Core temperature was monitored by a thermocouple probe (TH-212, Bjhocy science and technology Co. Ltd., Beijing, China) that was placed into the esophagus and maintained with a heat lamp throughout the experiment to ensure appropriate temperature management. Left ventricular ejection fraction (LVEF) was noninvasively assessed at baseline and at hourly intervals after resuscitation with an echocardiograph system (DC-6, Mindray Medical International Limited, Shenzhen, China).

Arterial blood samples were drawn at baseline and, 3 and 6 h after ROSC. Blood gases were measured with the aid of a blood analyzer (i-STAT, Abbott Point of Care Inc, Abbott Park, IL, USA). Serum concentrations of cardiac troponin T (cTnT) and S100B that were quantified with an enzyme-linked immunoassay (Elisa Kit, Cusbio Biotech Co. Ltd., Wuhan, China) according to the manufacturer’s instructions served as biomarkers of cardiac and cerebral injury, respectively^17^.

The neurological deficit score (NDS) was examined 24, 48, 72 and 96 h after resuscitation and confirmed by 2 investigators blinded to the treatment. The level of consciousness and breathing, the cornea reflex, cranial nerve reflexes, the auditory reflex, motor sensory function, and coordination behavior were scored according to a standardized NDS system (0 to 500 scale; 0: no observed neurological deficit, 500: death or brain death) that was developed to evaluate neurological outcome after global cerebral ischemia for rats^18^. The NDS has been shown to be positively correlated with global histological damage and has been adopted in rodent animal studies to assess behavioral performance and neurological recovery after cardiac arrest and CPR^14,16,19^.

After 96 h of NDS evaluation, the surviving animals were euthanized with sodium pentobarbital. The brains were removed and immersed in paraformaldehyde. The paraffin-embedded brains were sliced in 10 µm sections and then stained with hematoxylin and eosin. Each section was visually assessed according to the histopathologic damage score (HDS) in the CA1 region of the hippocampus under a microscope at 400 × magnification^20^. The degenerated neurons were discerned from the viable ones by examining the morphological changes, i.e., pyknosis, karyorrhexis, karyolysis and cytoplasmic changes; the HDS was graded as 1 (<25%), 2 (25% to 50%), 3 (50% to 75%), and 4 (>75%), respectively, based on the percentage of each visual field occupied by the degenerated neurons^20–22^.

### Statistical analyses

The normal distribution of the data was confirmed using the Shapiro-Wilk test. Data are reported as the means with SDs or medians including interquartile ranges. Hemodynamic and biochemical variables were compared by a general linear model for repeated measures analyses, followed by ANOVA with Bonferroni correction for post hoc comparisons. Physiological variables were examined by repeated-measures analysis comprising treatment group, time, and treatment-by-time interaction. NDS data were analyzed with the Kruskal-Wallis nonparametric test followed by a post hoc Mann-Whitney test. Fisher’s exact test was performed for categorical data. The Kaplan-Meier analysis and the log-rank test were used for survival analysis and comparisons. A *p* < 0.05 was regarded as statistically significant.

## Results

The baseline physiological measurements and characteristics of CPR did not differ significantly among the 5 groups (Table 1). All animals were successfully resuscitated and survived the 6 h postresuscitation monitoring period except two in the NNC group.

**Table 1:** Baseline variables and characteristics of CPR

Group	NNC	O_2__0h	O_2__1h	O_2__3h	O_2__5h
Body weight, g	312.1 ± 21.5	319.8 ± 27.9	313.1 ± 35.2	311.4 ± 34.7	313.9 ± 30.3
Heart rate, bpm	412.0 ± 28.1	420.6 ± 36.7	413.3 ± 22.7	428.0 ± 25.1	422.4 ± 27.5
MAP, mmHg	125.8 ± 8.1	121.4 ± 10.9	118.7 ± 16.2	122.8 ± 8.8	122.2 ± 11.2
Temperature, °C	37.5 ± 0.1	37.5 ± 0.3	37.5 ± 0.4	37.4 ± 0.3	37.5 ± 0.2
LVEF, %	78.5 ± 5.9	77.9 ± 4.1	77.7 ± 6.3	77.0 ± 5.9	78.5 ± 5.7
Asphyxial time to cardiac arrest, s	165.3 ± 15.6	172.3 ± 11.8	168.9 ± 13.6	169.8 ± 16.7	170.1 ± 17.3
CPR duration, s	75.5 ± 34.4	73.1 ± 15.6	79.6 ± 23.1	74.2 ± 33.0	82.7 ± 29.8
Number of shocks, n	0.3 ± 0.4	0.2 ± 0.5	0.4 ± 0.6	0.4 ± 0.6	0.4 ± 0.5
Total epinephrine, µg	7.1 ± 2.4	6.5 ± 1.3	6.2 ± 2.0	6.2 ± 2.0	6.8 ± 1.3
ROSC rate, %	100	100	100	100	100

CPR: cardiopulmonary resuscitation; NNC: normoxic control under normothermia; O_2__0h, O_2__1h, O_2__3h, and O_2__5h: ventilated with 100% oxygen for 0, 1, 3 and 5 h under targeted temperature management; bpm: beats per minute; MAP: mean arterial pressure; LVEF: left ventricular ejection fraction; ROSC: return of spontaneous circulation. n = 16 in each group.

**Figure 1:**
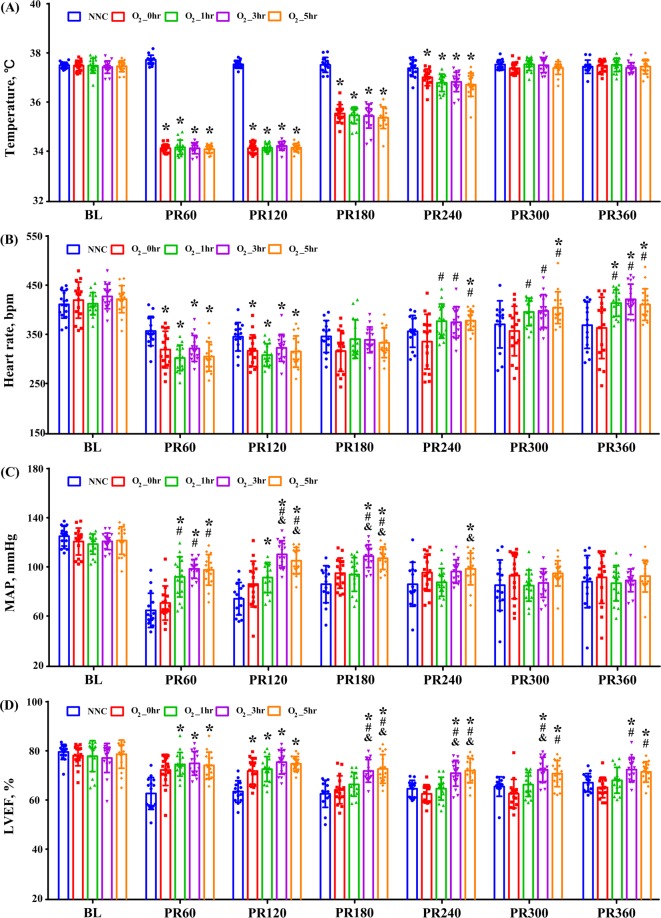
Esophageal temperature (**A**), heart rate (**B**), mean arterial pressure (MAP) (**C**), and left ventricular ejection fraction (**D**) measurements before and after cardiac arrest. BL: baseline; PR: postresuscitation; bpm: beats per minute; NNC: normoxic control under normothermia; O_2__0h, O_2__1h, O_2__3h, and O_2__5h: ventilated with 100% oxygen for 0, 1, 3 and 5 h under targeted temperature management; *p < 0.05 compared with NNC; ^#^*p* < 0.05 com*p*ared with O_2__0h. ^&^*p* < 0.05 compared with O_2__1h. n = 16 at each time point except for n = 15 and 14 at 60 and 120~360 minutes in the NNC group.

Figure 1 shows the hemodynamic data measured during the postresuscitation phase and no differences in temperature (Fig. 1A) were observed among the 4 experimental groups at each time point. The heart rate was significantly lower during hypothermia in all of the experimental groups but was considerably higher in the O_2__1h, O_2__3h and O_2__5h groups after rewarming to normothermia (Fig. 1B). MAP measured at 60 min postresuscitation in the O_2__1h, O_2__3h and O_2__5h groups, measured at 120 and 180 min in the O_2__3h and O_2__5h groups and measured at 240 in the O_2__5h group was markedly higher among groups (Fig. 1C). Compared with the NNC group, all 4 experimental groups demonstrated significantly higher LVEF during the first 2 h but was only preserved in the O_2__3h and O_2__5h groups after that (Fig. 1D).

The arterial blood gas measurements are listed in Table 2. One hour after ROSC, arterial oxygen saturation (SaO_2_), partial pressure of arterial oxygen (PaO_2_) and lactate measurements were significantly higher in the O_2__1h, O_2__3h and O_2__5h groups than in the NNC and O_2__0h groups. Three hours after resuscitation, SaO_2_ and PaO_2_ were significantly higher in the O_2__3h and O_2__5h groups than in the NNC, O_2__0h and O_2__1h groups. At the same time, the partial pressure of arterial carbon dioxide (PaCO_2_) was markedly higher in the O_2__3h and O_2__5h groups than in the NNC and O_2__1h group. Six hours after ROSC, SaO_2_ was significantly higher in the O_2__5h group, PaO_2_ was significantly higher in the O_2__1h, O_2__3h and O_2__5h groups, PaCO_2_ was significantly higher in the O_2__3h group and lactate was considerably lower in the O_2__3h and O_2__5h groups.

**Table 2:** Arterial blood gas analyses at baseline and 60, 180 and 360 min (PR60, PR180, and PR360) after resuscitation

Group	Baseline	PR60	PR180	PR360
SaO_2_, %				
NNC	94.4 ± 2.0	88.4 ± 4.0	95.5 ± 2.1	93.6 ± 2.1
O_2__0h	94.3 ± 1.8	84.4 ± 5.0*	94.2 ± 3.0	96.5 ± 1.4*
O_2__1h	94.9 ± 1.5	98.1 ± 2.0*^#^	96.9 ± 1.1*^#^	96.6 ± 1.5*
O_2__3h	94.4 ± 2.8	98.2 ± 2.6*^#^	99.1 ± 1.0*^#&^	95.7 ± 1.4*
O_2__5h	94.2 ± 3.2	98.6 ± 1.0*^#^	99.4 ± 1.0*^#&^	97.7 ± 0.6*^#&^
PaO_2_, mmHg				
NNC	73.2 ± 3.8	62.9 ± 8.7	80.7 ± 8.1	77.3 ± 12.3
O_2__0h	74.2 ± 6.8	58.2 ± 6.8	78.1 ± 12.6	89.4 ± 8.4*
O_2__1h	78.4 ± 6.7	149.9 ± 34.0*^#^	93.9 ± 8.7*^#^	97.4 ± 8.7*^#^
O_2__3h	76.4 ± 8.7	146.1 ± 33.9*^#^	175.3 ± 16.0*^#&^	98.4 ± 9.5*^#^
O_2__5h	75.7 ± 6.9	155.1 ± 19.4*^#^	177.6 ± 17.5*^#&^	96.4 ± 6.8*^#^
PaCO_2_, mmHg				
NNC	38.7 ± 4.1	33.7 ± 4.8	31.3 ± 6.9	34.5 ± 4.0
O_2__0h	38.6 ± 4.7	41.0 ± 5.2*	36.4 ± 5.7*	33.5 ± 4.3
O_2__1h	37.8 ± 4.5	38.4 ± 4.6*	33.4 ± 4.3	32.7 ± 3.6
O_2__3h	38.0 ± 4.1	37.7 ± 4.5*	38.0 ± 5.0*&	37.4 ± 4.1#&
O_2__5h	37.1 ± 4.0	38.3 ± 4.8*	37.3 ± 4.0*&	34.7 ± 4.0
PH				
NNC	7.42 ± 0.05	7.32 ± 0.09	7.39 ± 0.06	7.36 ± 0.05
O_2__0h	7.41 ± 0.03	7.24 ± 0.05*	7.33 ± 0.07*	7.38 ± 0.17
O_2__1h	7.41 ± 0.05	7.29 ± 0.04#	7.32 ± 0.05*	7.41 ± 0.11
O_2__3h	7.42 ± 0.03	7.28 ± 0.04#	7.32 ± 0.06*	7.38 ± 0.06
O_2__5h	7.41 ± 0.04	7.31 ± 0.06#	7.35 ± 0.07*	7.41 ± 0.06*
Lactate, mmol/l				
NNC	0.87 ± 0.31	3.83 ± 1.20	1.64 ± 0.57	1.59 ± 0.56
O_2__0h	0.89 ± 0.40	4.36 ± 0.80	2.04 ± 0.60*	1.71 ± 0.55
O_2__1h	0.86 ± 0.37	2.23 ± 0.81*^#^	1.71 ± 0.37	1.57 ± 0.37
O_2__3h	0.86 ± 0.29	2.28 ± 0.76*^#^	1.41 ± 0.29^#&^	0.98 ± 0.51^#&^
O_2__5h	0.85 ± 0.38	2.32 ± 0.67*^#^	1.33 ± 0.31^#&^	1.02 ± 0.42^#&^

NNC: normoxic control under normothermia; O_2__0h, O_2__1h, O_2__3h, and O_2__5h: ventilated with 100% oxygen for 0, 1, 3 and 5 h under targeted temperature management; SaO_2_, arterial oxygen saturation; PaO_2_, partial pressure of arterial oxygen; PaCO_2_, partial pressure of arterial carbon dioxide. *p < 0.05 compared with NNC; ^#^*p* < 0.05 com*p*ared with O_2__0h; ^&^*p* < 0.05 compared with O_2__1h. n = 16 at each time point except for n = 14, 14 and 13 at 60, 180 and 360 minutes in the NNC group.

**Figure 2:**
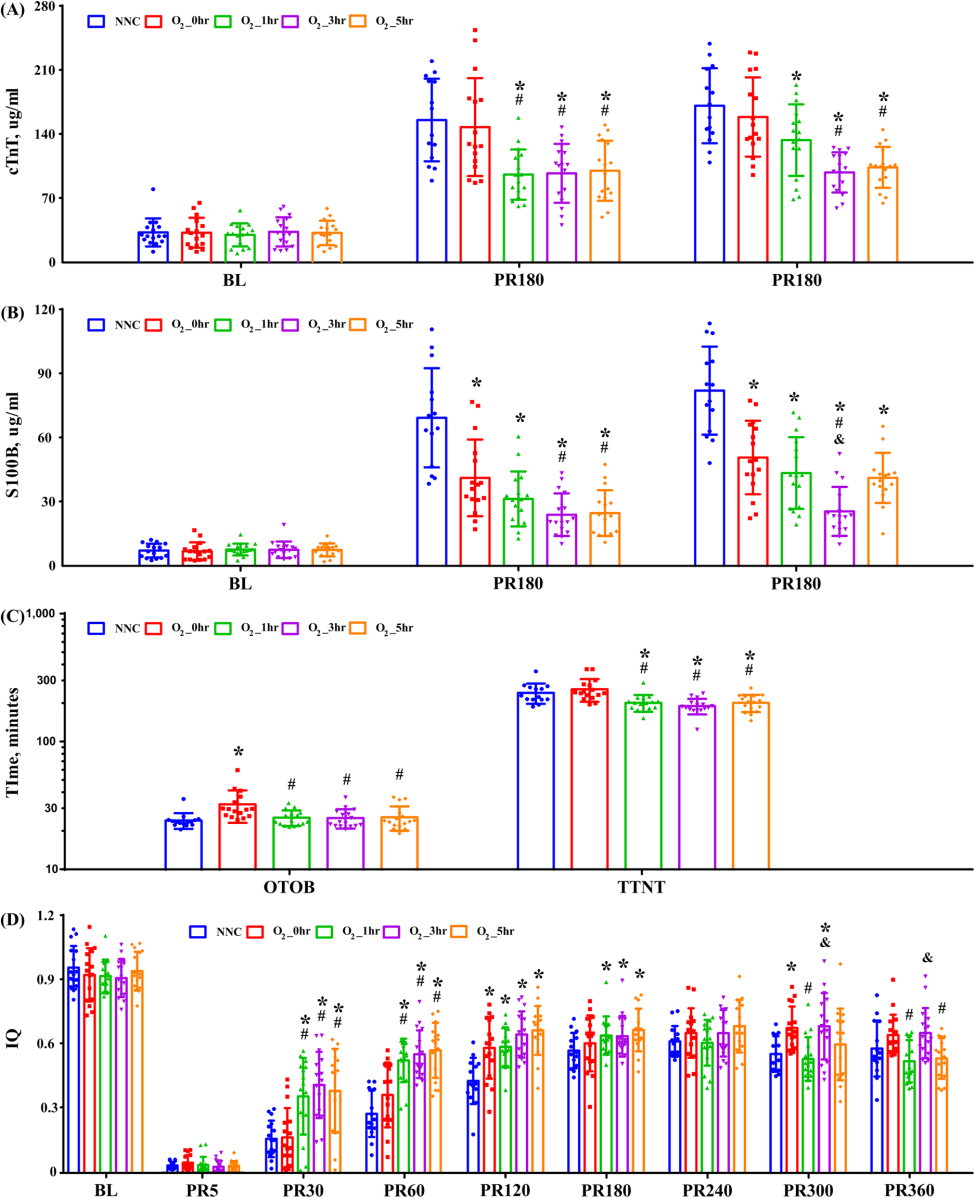
Circulating levels of cardiac troponin T (cTnT) (**A**) and serum S100B (**B**), the onset time of the EEG burst (OTOB), the time to normal EEG trace (TTNT) (**C**), and information quantity (IQ) of EEG (**D**) in the experimental groups. PR180 and PR360: 180 and 360 min postresuscitation; NNC: normoxic control under normothermia; O_2__0h, O_2__1h, O_2__3h, and O_2__5h: ventilated with 100% oxygen for 0, 1, 3 and 5 h under targeted temperature management; *p < 0.05 compared with NNC; ^#^*p* < 0.05 com*p*ared with O_2__0h; ^&^*p* < 0.05 compared with O_2__1h. n = 16 at each time point except for n = 15 and 14 at 60 and 120~360 minutes in the NNC group.

Figure 2 shows the measured serum cTnT and S100B, EEG OTOB, TTNT and IQ. Both cTnT and S100B were dramatically increased after resuscitation. Compared with the NNC and O_2__0h groups, the O_2__3h and O_2__5h groups demonstrated significantly lower serum cTnT levels after ROSC(Fig. 2A). At the same time, serum S100B levels in the TTM groups were significantly lower than those in the NNC group. In addition, the S100B measurement in the O_2__3h group was substantially lower than that in the other 3 experimental groups (Fig. 2B). The OTOB was considerably longer in the O_2__0h group, while TTNT was considerably shorter in the O_2__1h, O_2__3h and O_2__5h groups (Fig. 2C). IQ measured at 30 and 60 min after ROSC in the O_2__1h, O_2__3h and O_2__5h groups was significantly higher than that measured in the NNC and O_2__0h groups (Fig. 2D). In addition, IQ measured at 300 and 360 min in the O_2__3h groups was relatively higher than that measured in the other groups.

**Figure 3:**
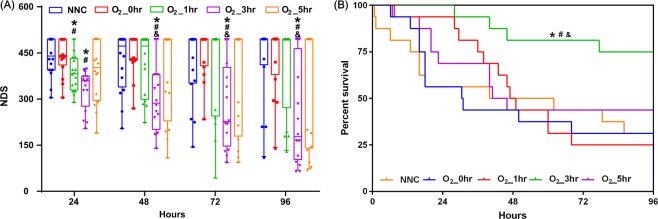
Neurologic deficit score (NDS) (**A**) and Kaplan-Meier survival curve (**B**) in the experimental groups. NNC: normoxic control under normothermia; O_2__0h, O_2__1h, O_2__3h, and O_2__5h: ventilated with 100% oxygen for 0, 1, 3 and 5 h under targeted temperature management; *p < 0.05 compared with NNC; ^#^*p* < 0.05 com*p*ared with O_2__0h; ^&^*p* < 0.05 compared with O_2__1h. n = 16 in each group.

The NDSs measured during the 4 days following resuscitation are listed in Fig. 3A. The NDSs measured at 24 h in the O_2__1h and O_2__3h groups were significantly lower than those measured in the NNC and O_2__0h groups. Thereafter, the NDS values were significantly lower in the O_2__3h group than in the NNC, O_2__0h and O_2__1h groups. Five animals in the NNC group, 5 in the O_2__0h group, 4 in the O_2__1h group, 12 in the O_2__3h group and 7 in the O_2__5h group survived to 96 h. The cumulative 96 h survival rate was significantly higher in the O_2__3h group than in the NNC (75.0% vs. 31.3%, *p* = 0.008), O_2__0h (75.0% vs. 31.3%, *p* = 0.014), O_2__1h (75.0% vs. 25.0%, *p* = 0.011) and O_2__5h (75.0% vs. 43.8%, *p* = 0.038) groups (Fig. 3B). There were no significant differences in survival rate among the NNC, O_2__0h, O_2__1h and O_2__5h groups.

**Figure 4:**
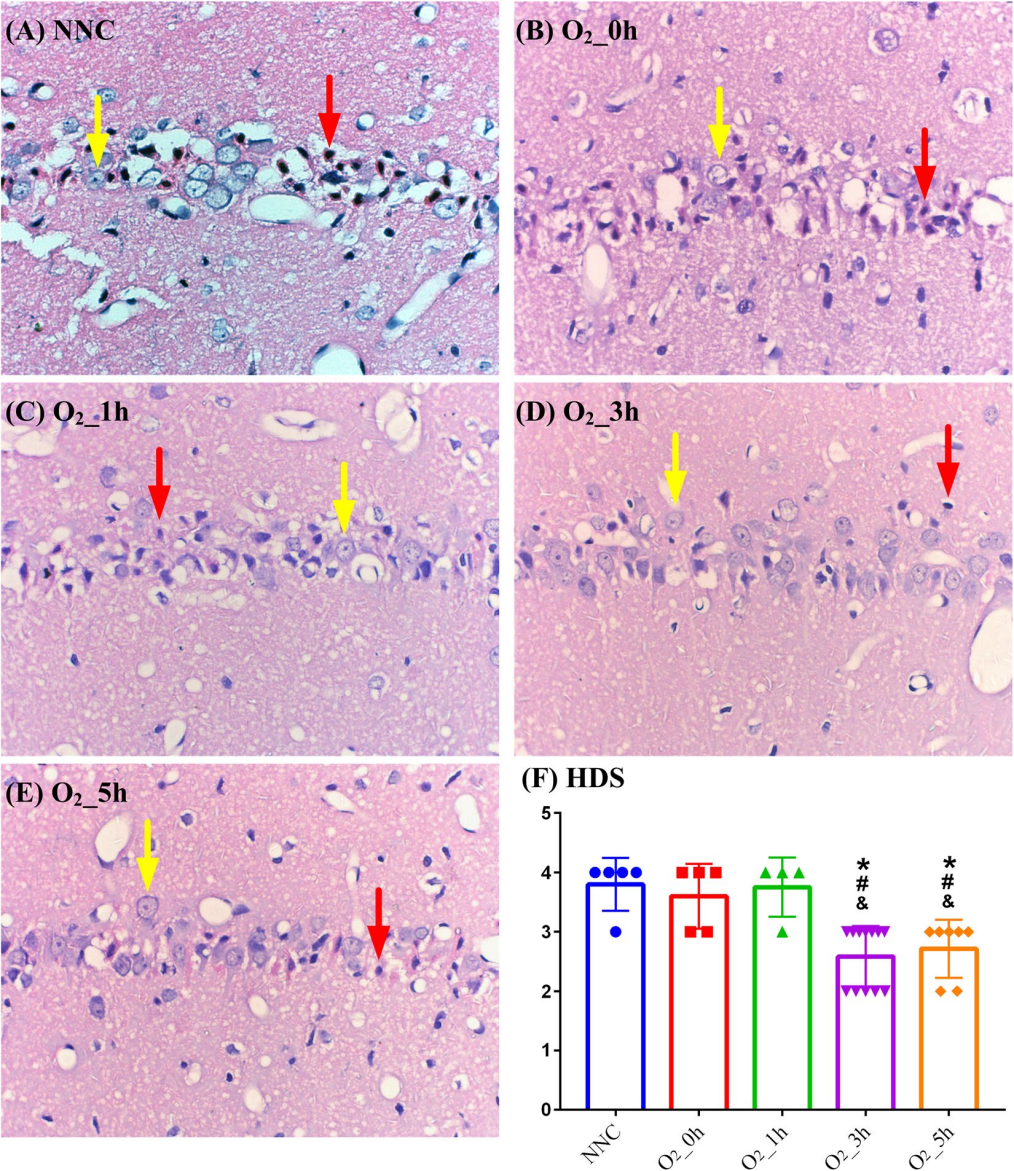
Representative micrographs (magnification, ×400) of the hematoxylin and eosin-stained CA1 region of the hippocampus 96 h after resuscitation in the surviving animals (**A–E**) and the overall HDS (**F**). Yellow arrows indicate viable neurons, and red arrows indicate degenerated neurons. NNC: normoxic control under normothermia; O_2__0h, O_2__1h, O_2__3h, and O_2__5h: ventilated with 100% oxygen for 0, 1, 3 and 5 h under targeted temperature management; HDS: histopathologic damage score. n = 5, 5, 4, 12 and 7 in the NNC, O_2__0h, O_2__1h, O_2__3h, and O_2__5 h group respectively.

Figure 4 shows representative sections from the CA1 region of the hippocampus. The number of degenerated neurons was significantly lower in the O_2__3h and O_2__5h groups than in the O_2__0h and O_2__1h groups. The overall HDSs were markedly lower in the O_2__3h (2.58 ± 0.51 vs. 3.80 ± 0.45, *p* < 0.01, 2.58 ± 0.51 vs. 3.60 ± 0.55, *p* = 0.01, 2.58 ± 0.51 vs. 3.75 ± 0.43, *p* = 0.01) and O_2__5h (2.71 ± 0.49 vs. 3.80 ± 0.45, *p* < 0.01, 2.71 ± 0.49 vs. 3.60 ± 0.55, *p* = 0.02, 2.71 ± 0.49 vs. 3.75 ± 0.43, *p* = 0.01) groups compared with the NNC (3.80 ± 0.45), O_2__0h (3.60 ± 0.55) and O_2__1h (3.75 ± 0.43) groups (Fig. 4F).

## Discussion

The present study addressed the effects of the duration of 100% postresuscitation oxygen administration on neurological outcome and survival in an asphyxial cardiac arrest rat model. Our results indicate that the duration of hyperoxic ventilation after resuscitation impacts neurological recovery in animals treated with TTM, and inhaling high concentrations of oxygen during hypothermia greatly improves 96 h survival from asphyxial cardiac arrest.

Cardiac arrest causes an abrupt cessation in the delivery of oxygen and cerebral hypoxia occurs when there is not sufficient oxygen supplied to the brain. Administering high concentrations (100%) of oxygen during CPR is advised because it may facilitate ROSC^23^. However, there has been recent debate over this practice during the early postresuscitation stages, with concerns that exposure to high concentrations of oxygen may exacerbate ischemia-reperfusion injury^24^. Experimental animal studies assessing the effects of high-concentration oxygen administration unanimously have suggested that inhalation of 100% oxygen is associated with aggravated brain damage and/or worse neurological outcome^9^, except two studies that reported that hyperoxic ventilation did not affect outcome^25,26^. Considering that the post cardiac arrest syndrome is a continuous process, the within 60 min time course of 100% oxygen administration in these animal studies may be too low to have an impact. Moreover, no TTM was applied in the abovementioned studies, so the observations cannot be readily translated to humans. Clinical studies have regrettably reported conflicting results regarding postresuscitation oxygen management. Kilgannon *et al*. reported that hyperoxia in arteries was independently associated with the high in-hospital mortality in patients without TTM^27^. This result was supported by subsequent clinical studies in patients treated with TTM^11,12,28,29^. By contrast, Bellomo *et al*. demonstrated that postresuscitation hyperoxia did not affect prognosis when confounding factors were adjusted in a large database^30^. This result was also supported by subsequent clinical studies, including patients resuscitated from both shockable and non-shockable rhythms^31–35^. Furthermore, Wang and Vaahersalo found that PaO_2_ values of patients with good neurological prognosis was higher than that of patients with poor neurological prognosis^10,36^. These conflicting conclusions can be explained by the heterogeneity of these studies. On the one hand, the cause of cardiac arrest, presenting heart rhythm, duration of CPR and postresuscitation TTM are confounders that may impact neurological outcomes. One the other hand, most previous studies evaluated PaO_2_ measurements over the first 24 h after ROSC and did not control the time course of hyperoxic ventilation.

This is the first study to assess the impact of duration of high-concentration oxygen administration on neurological outcome in a model of cardiac arrest in rats treated with TTM. Although hypothermia alleviated brain damage in the early stage of postresuscitation, there was no significant improvement in neurological function and survival after 96 h in animals treated with hypothermia. This result is consistent with previous studies on hypothermic effects in animal models of asphyxiated cardiac arrest^37,38^, but contradicts those in animal models of ventricular fibrillation^16^. The discrepancy can be explained by the difference in the mechanism of brain damage between the two most prevalent causes of cardiac arrest. Compared with ventricular fibrillation, asphyxia causes more severe brain damage and may require different cerebral resuscitation treatments^14^. To improve the prognosis of asphyxial cardiac arrest, postresuscitation TTM may need to be combined with other approaches, such as hyperoxic ventilation.

Our results suggest that postresuscitation hyperoxic ventilation improves hemodynamics when combined with TTM. Compared with the animals ventilated with normoxia, the animals that underwent hyperoxic ventilation demonstrated significantly improved MAP, heart rate and LVEF in a duration-dependent manners. Compared with normoxic ventilation, inhaling 100% oxygen for 1 h greatly improved MAP and heart rate but did not have an effect on LVEF. Additionally, prolonging 100% oxygen inhalation to 3 h significantly improved MAP and LVEF compared with inhalation of 100% oxygen for 1 h. However, prolonging 100% oxygen inhalation to 5 h did not further improve hemodynamics compared with inhalation of 100% oxygen for 3 h. The biochemical and histological results also suggested that postresuscitation hyperoxic ventilation mitigated myocardial and cerebral dysfunction, especially in animals administered 100% oxygen for 3 h. Consistently, quantitative parameters of EEG, which are prognostic indicators of neurological recovery, revealed that hyperoxic ventilation had an impact on prognostication. When neurological outcomes were measured, only animals administered 100% oxygen for 3 h showed improvements in NDS and survival.

The finding that optimal duration of high-concentration oxygen administration improves neurological outcome and survival can be attributed to the combined effects of oxygen and hypothermia. Maintaining adequate perfusion pressure is necessary for good neurological recovery because arterial hypotension is a common phenomenon in patients resuscitated from cardiac arrest and is correlated with the high in-hospital mortality^39^. Consistent with previous animal studies, inhaling 100% oxygen immediately after resuscitation greatly improved MAP in this study^40^. Improved MAP can affect brain blood flow, brain tissue oxygen delivery, and the course of ischemic-reperfusion brain injury after cardiac arrest^41,42^. Unfortunately, exposure to hyperoxia following resuscitation will also amplify the production of oxygen free radicals and promote free radical-generated damage contributing to cerebral injury and cardiac dysfunction^43,44^. However, the significantly reduced myocardial and cerebral dysfunction in animals ventilated with 100% oxygen during TTM indicate that concomitant hypothermia are involved in modifying the deleterious effect of hyperoxia. The physiological effects of TTM presumably is determined multifactorially, including the preservation of oxygen consumption and the suppression of free radical responses^45^. The toxic effects of breathing high concentrations of oxygen and the injuries associated with increased levels of oxidative stress may be mitigated by the beneficial effects of hypothermia. The successful balance between the disadvantages and benefits associated with hypoxia and hyperoxia thereby results in improved neurological outcomes^46^.

Our research has some limitations. First, we studied the single duration of cardiac arrest caused by asphyxia in healthy animals without complications. The effects of duration of postresuscitation hyperoxic ventilation still need to be evaluated in animal models of different causes of cardiac arrest, presenting rhythms as well as underlying comorbidities. Second, although we observed that hyperoxic ventilation during hypothermia improved neurological outcome, the relationship between this time course and the duration of untreated cardiac arrest and, hypothermia is still uncertain. Third, this was an observational study, and the effects of hyperoxic ventilation on brain tissue oxygen, oxidative stress, cell survival and proliferation, including the functional deficits of different regions, were not investigated. Fourth, in animal studies, hypothermia can be induced immediately after ROSC, and target temperature can be achieved within a few minutes. However, the induction time is typically up to a couple of hours in clinical studies. The optimal oxygen administration strategy during the induction of hypothermia is unknown.

## Conclusions

In this rat model of cardiac arrest, postresuscitation hyperoxic ventilation led to improved PaO_2_, PaCO_2_, hemodynamic, myocardial and cerebral recovery in animals treated with TTM. The beneficial effects of high-concentration oxygen were duration dependent, and ventilation with 100% oxygen during induced hypothermia contributed to improved neurological recovery and survival after 96 h. Our findings suggest that the precise control of postresuscitation high-concentration oxygen administration during TTM may avoid the harms associated with inadequate and excessive oxygenation.

## Supplementary information

Supplementary figure S1

Dataset 1
