# Supplementary material for: Effects of the duration of postresuscitation hyperoxic ventilation on neurological outcome and survival in an asphyxial cardiac arrest rat model
Source: Sci Rep. 2019 Nov 11;9:16500. doi: 10.1038/s41598-019-52477-y (PMC6848183; doi:10.1038/s41598-019-52477-y)
Supplement: Supplementary file 1 — Supplementary figure S1 [file 41598_2019_52477_MOESM1_ESM.pdf]

**Title:** Effects of the duration of postresuscitation hyperoxic ventilation on neurological outcome and survival in an asphyxial cardiac arrest rat model

**Authors:** Tongyi Hu[1], Jianjie Wang[1], Shuangwei Wang[2], Jingru Li[1], Bihua Chen[1], Feng Zuo[3], Lei Zhang[4], Yuanyuan Huang[5], Yongqin Li[1]

1 Department of Biomedical Engineering and Imaging Medicine, Army Medical University, Chongqing 400038, China

2 Shenzhen Dashen Institute of Biomedical Engineering Translation, Shenzhen 518060, China

3 Department of Information Technology, Southwest Hospital, Army Medical University, Chongqing 400038, China

4 Department of Emergency, Southwest Hospital, Army Medical University, Chongqing 400038, China

5 Department of Neurology, Southwest Hospital, Army Medical University, Chongqing 400038, China

**Address for correspondence:**

Yongqin Li, MSBME, PhD. Department of Biomedical Engineering and Imaging Medicine, Army Medical University. 30 Gaotanyan Main Street, Chongqing 400038, China.

Tel: (086) 23-6877-1725, Fax: (086) 23-6875-2329

E-mail: leeoken@gmail.com, leeoken@aliyun.com

**Supplementary figure S1:** The micrographs (magnification, ×400) of the hematoxylin and eosin-stained CA1 region of the hippocampus 96 h after resuscitation in the surviving animals

(A). normoxic control under normothermia (NNC) group

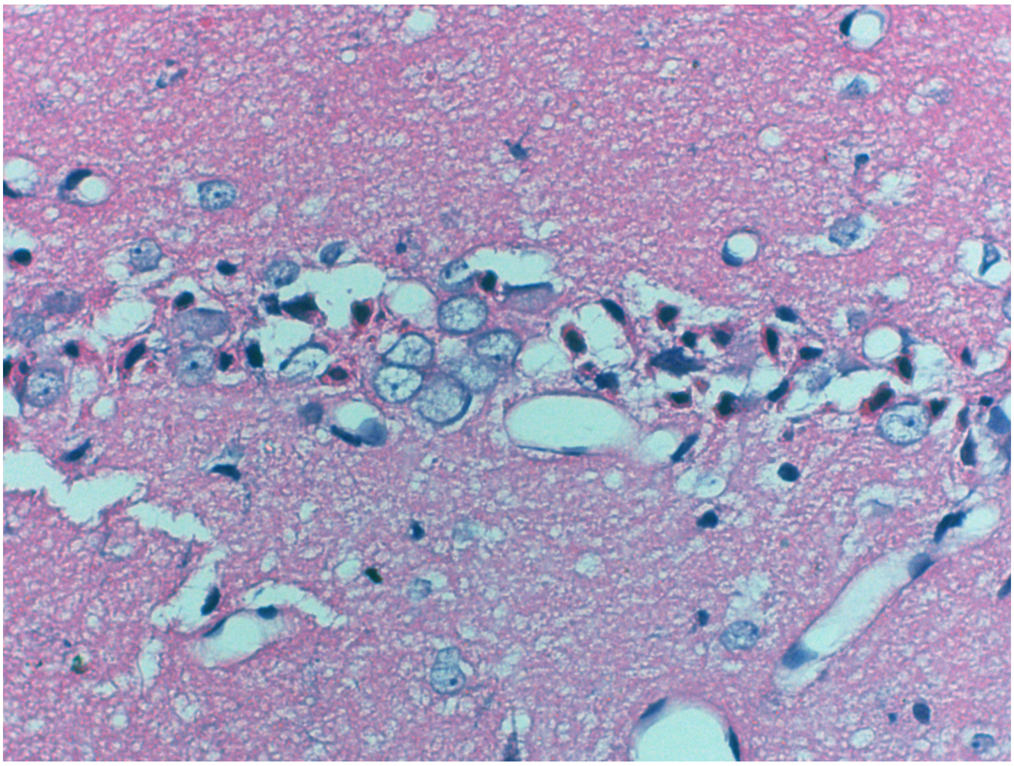

NNC_2

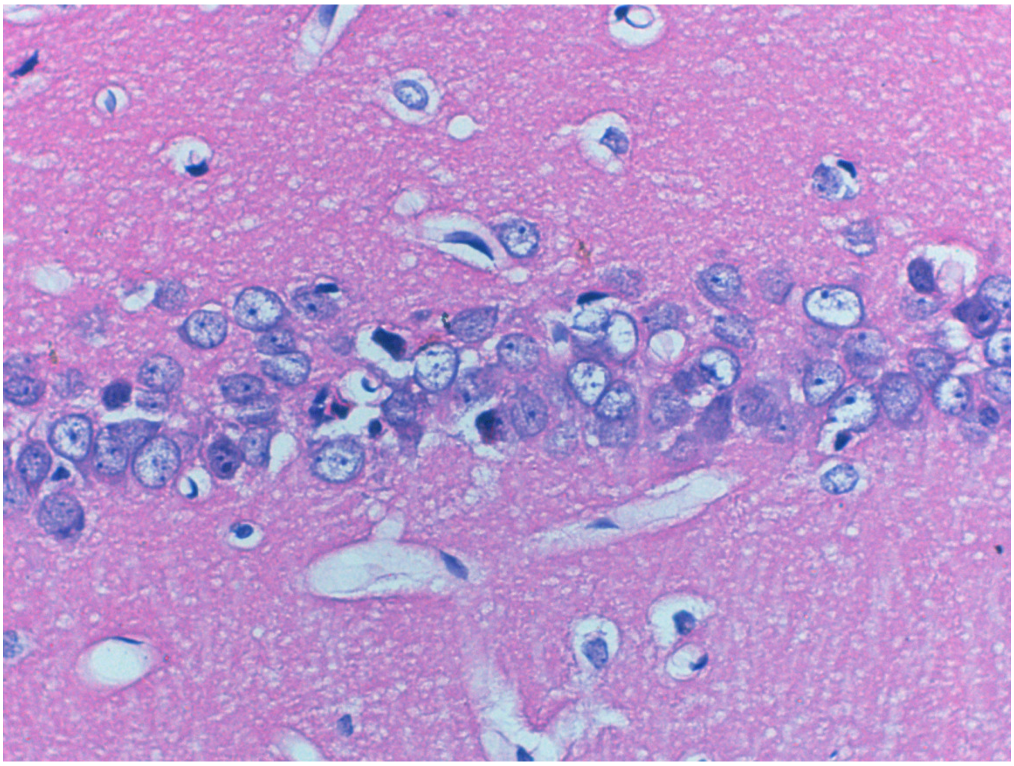

NNC_3

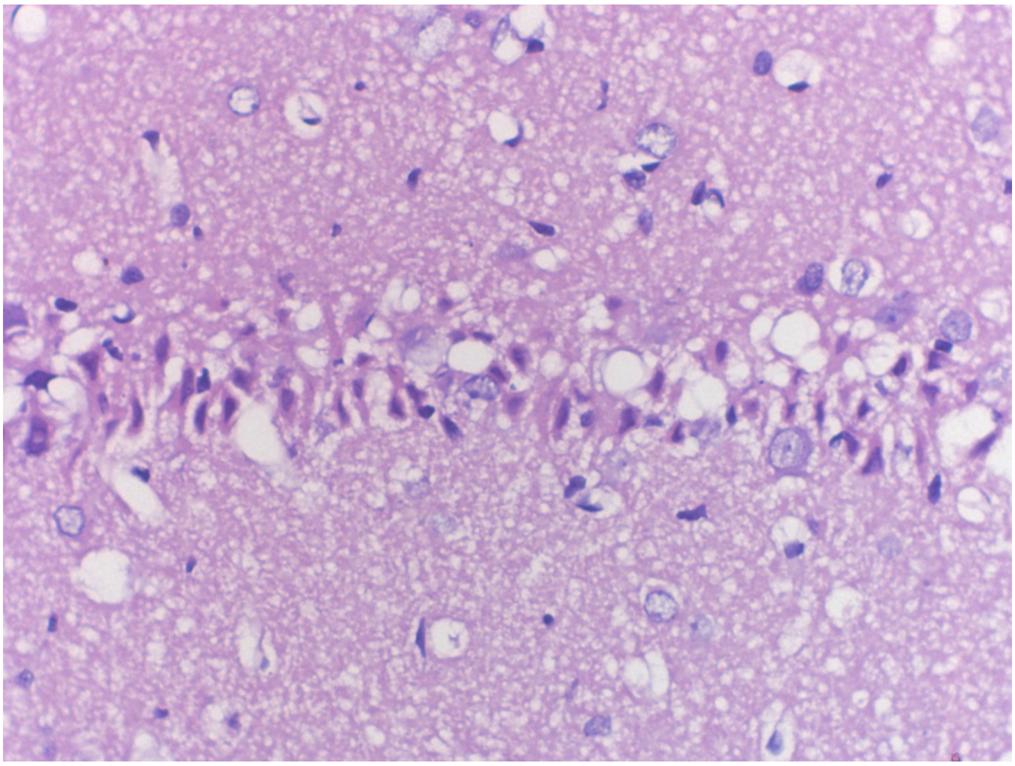

NNC_8

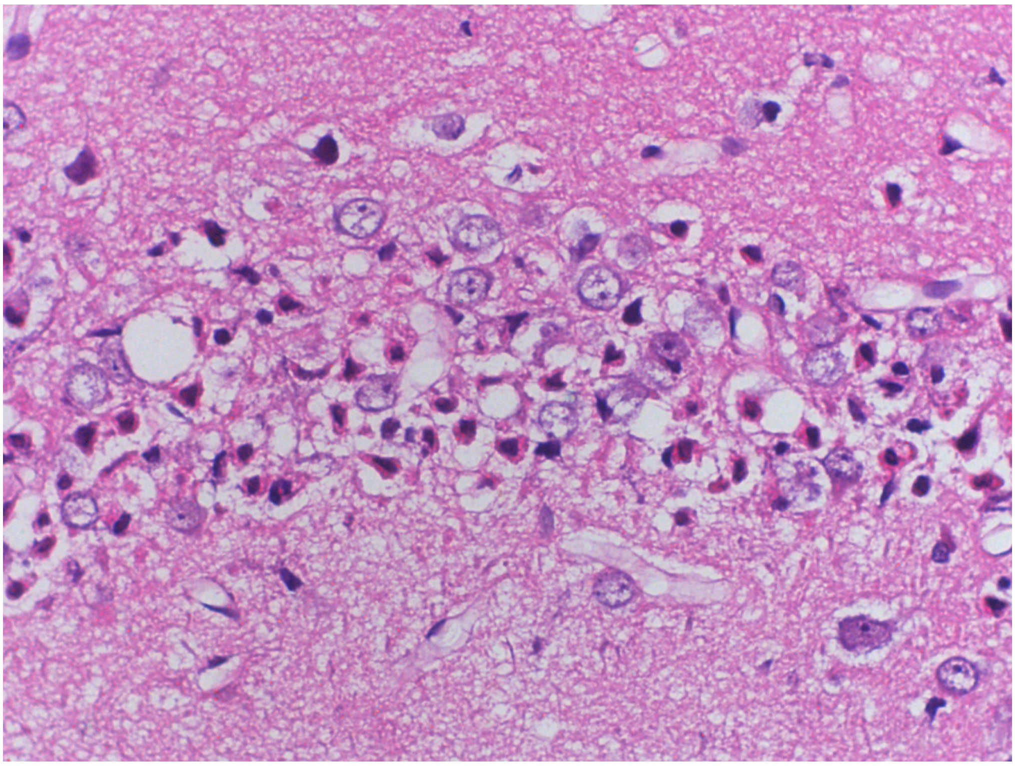

NNC_9

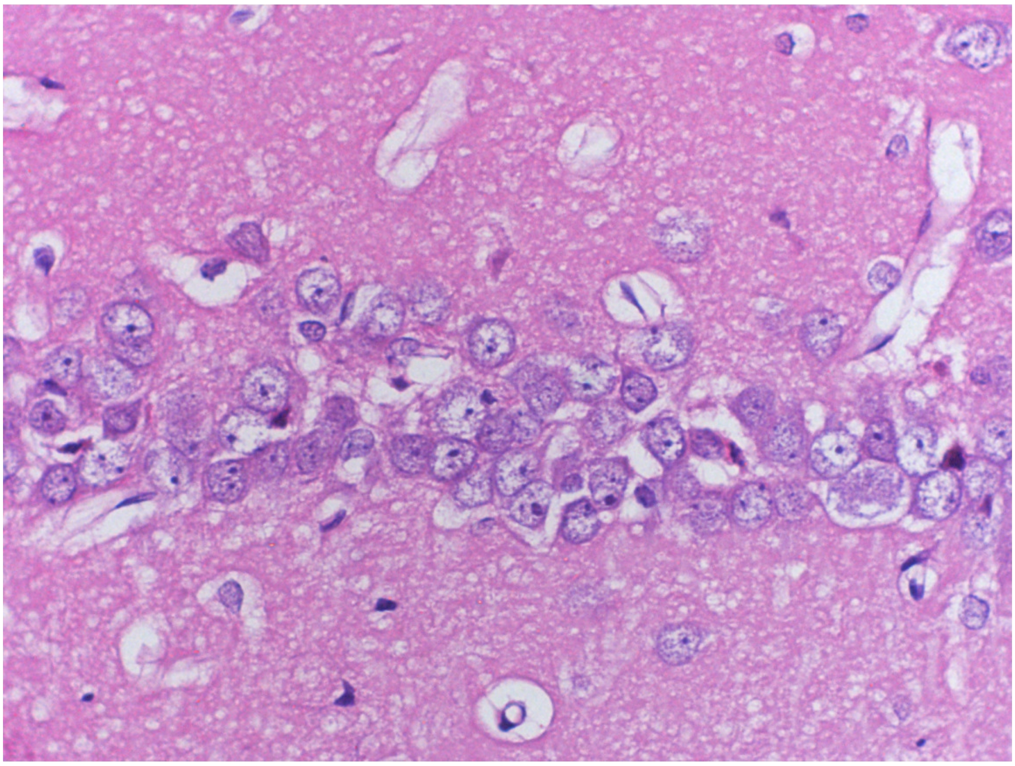

NNC_12

(B). ventilated with 100% oxygen for 0 ($O_2$_0h) hour group

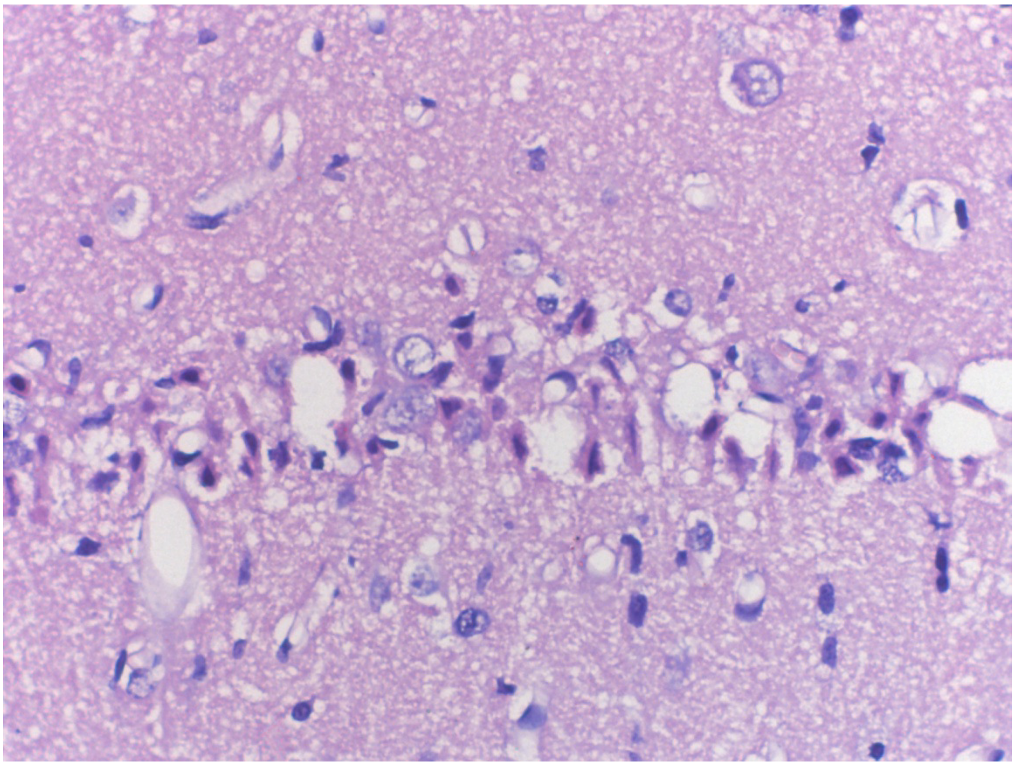

O2_0h_4

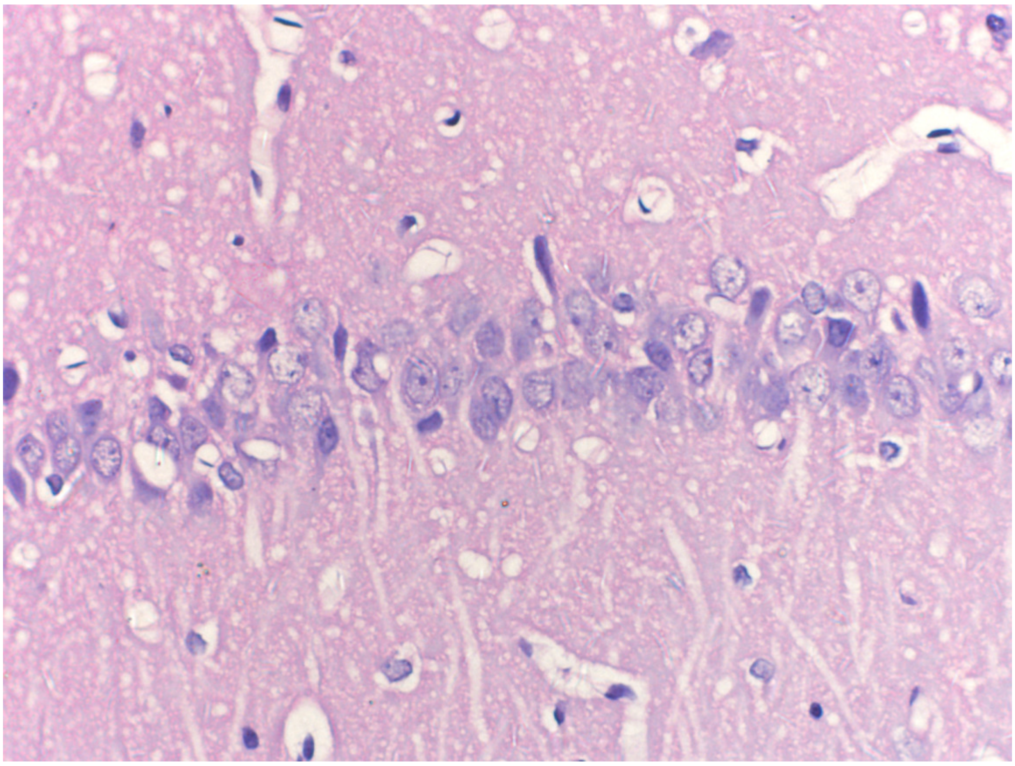

O2_0h_6

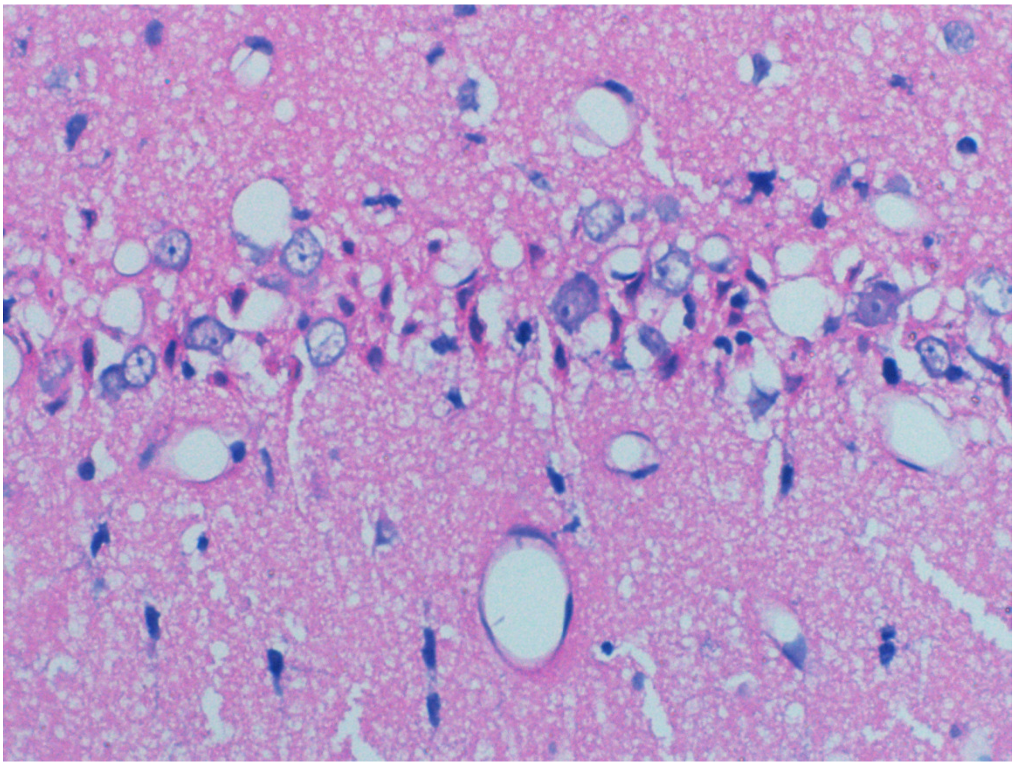

O2_0h_9

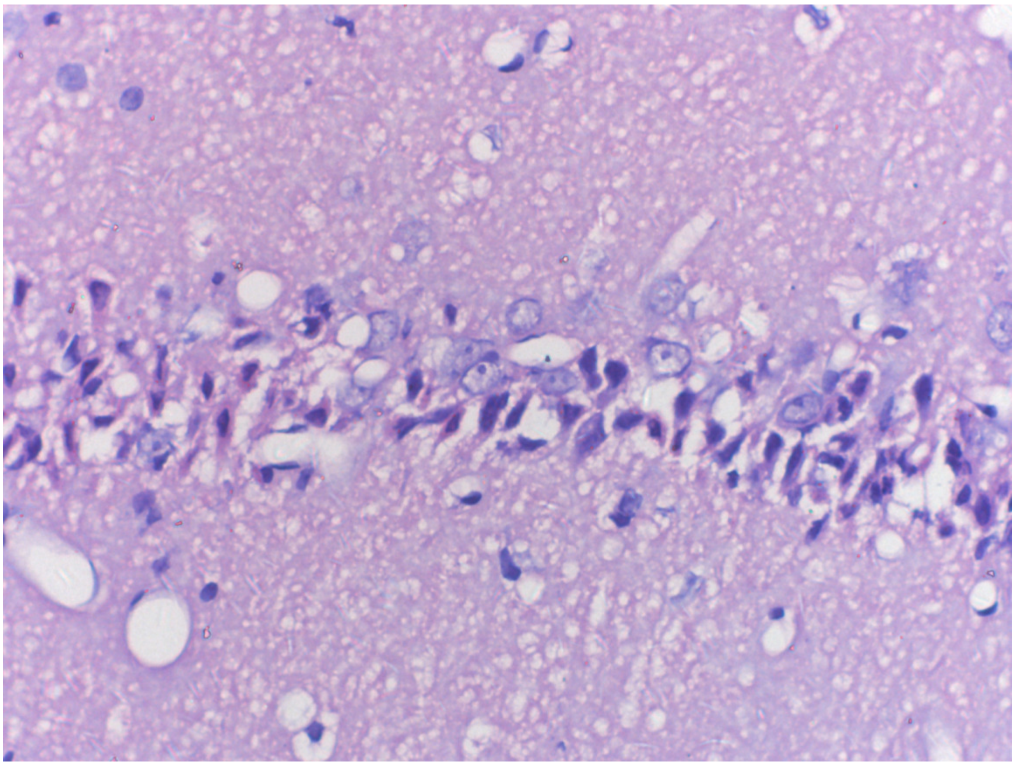

O2_0h_10

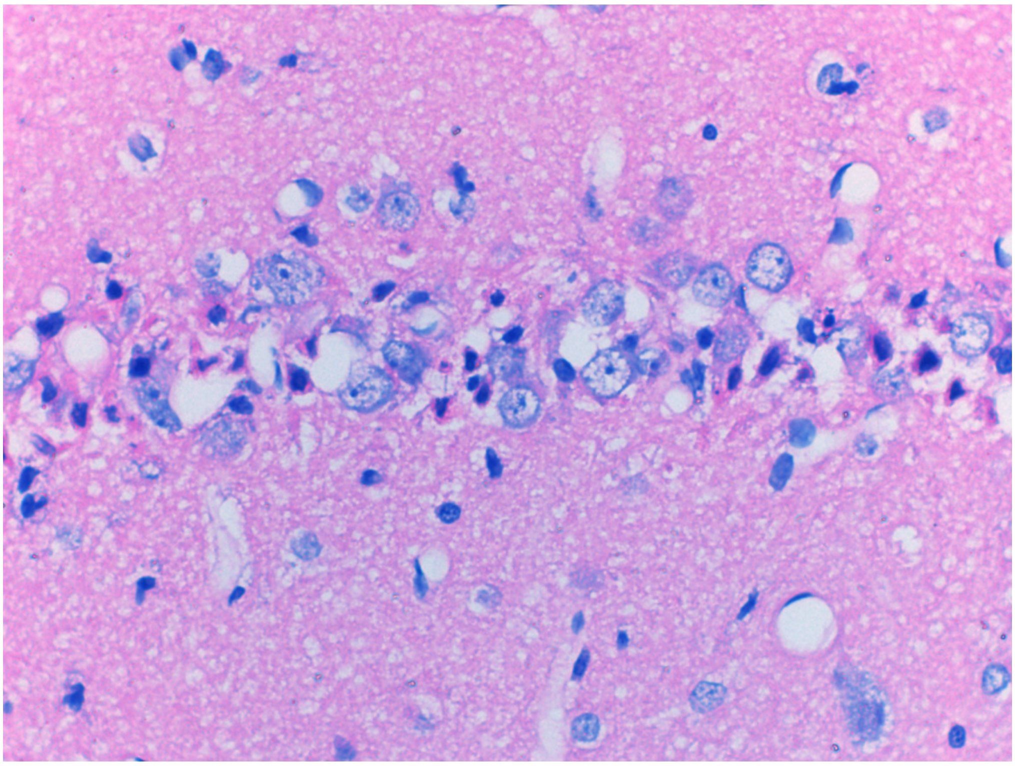

O2_0h_15

(C). ventilated with 100% oxygen for 1 ($O_2$_1h) hour group

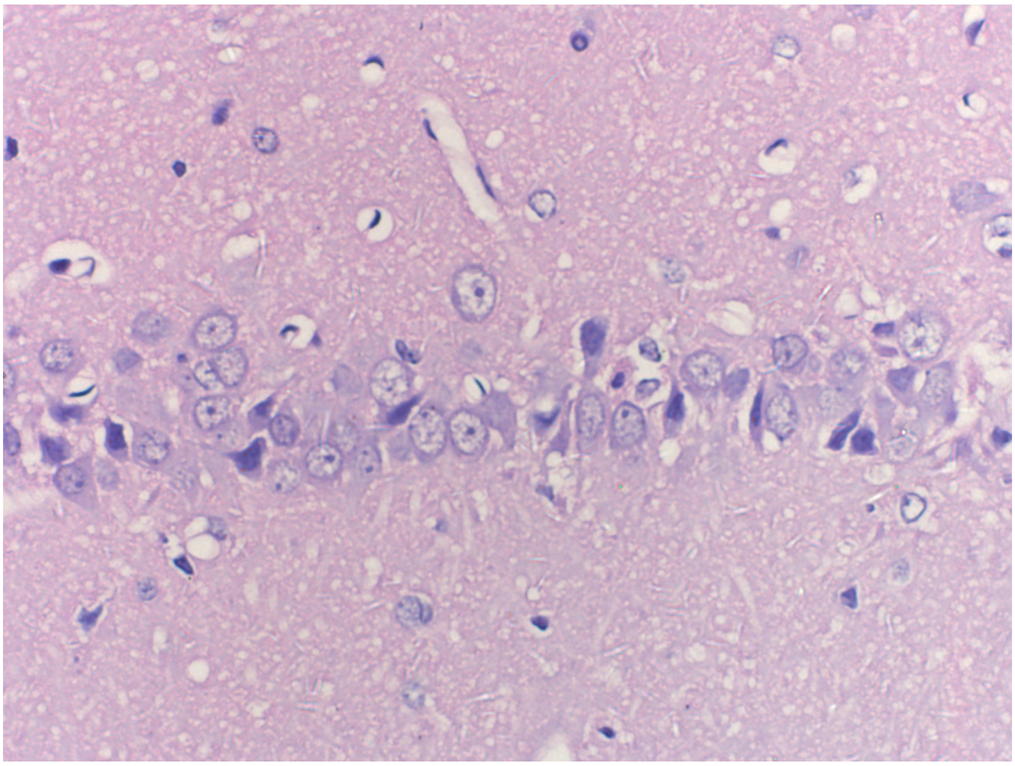

O2_1h_3

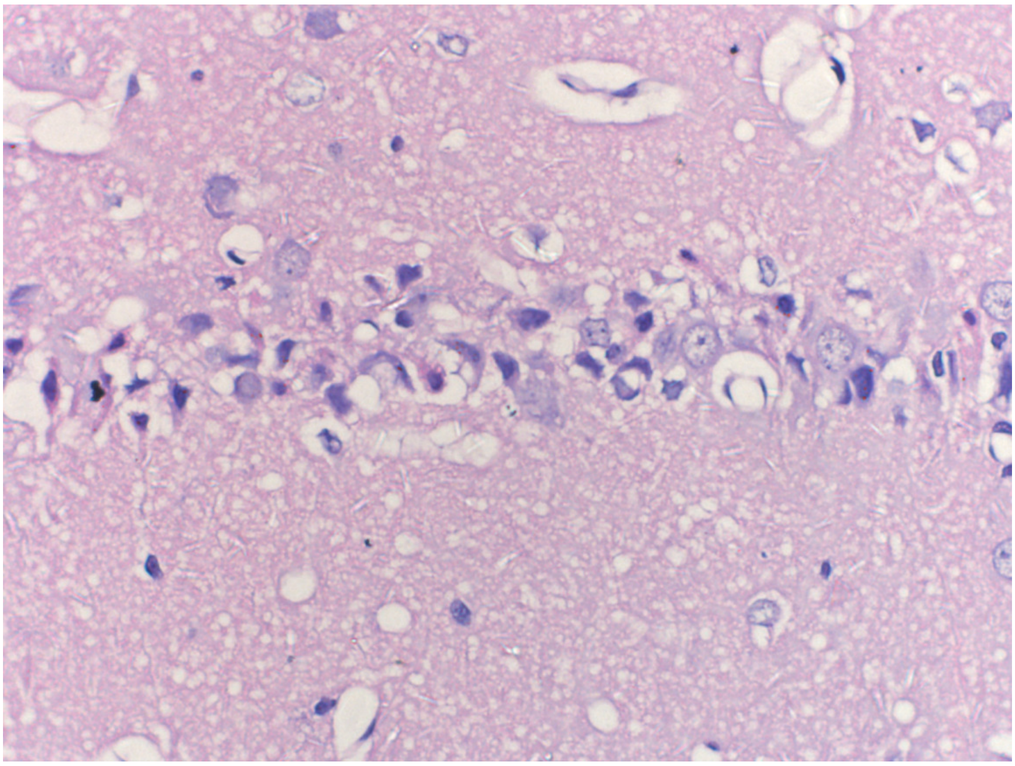

O2_1h_4

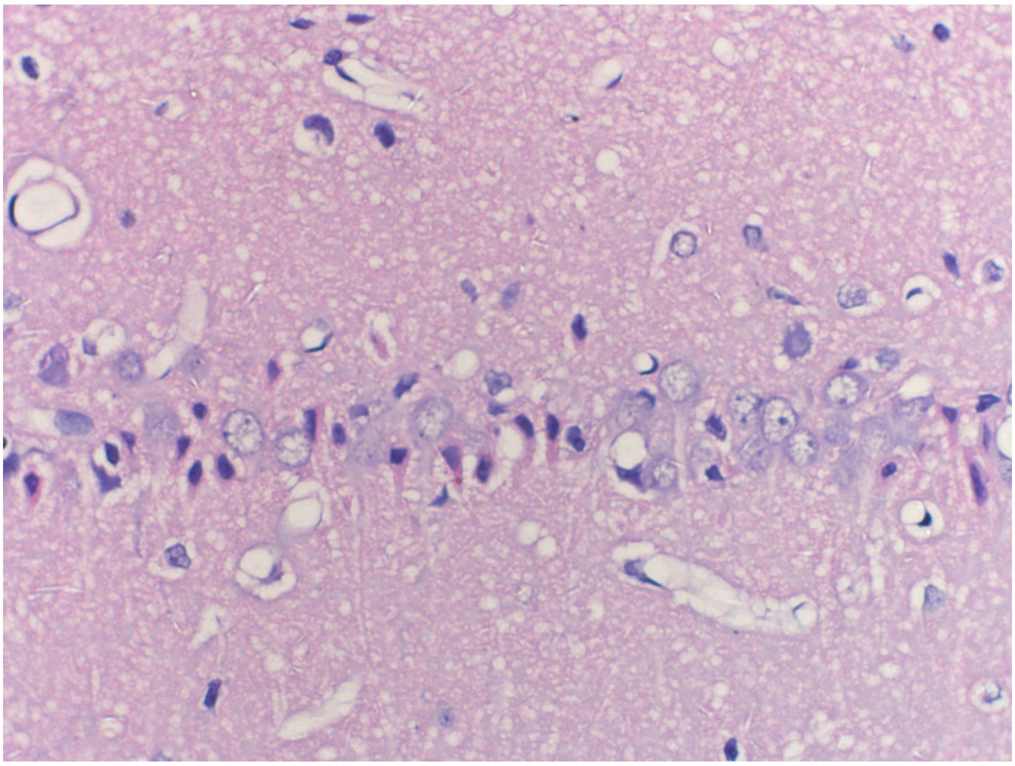

O2_1h_11

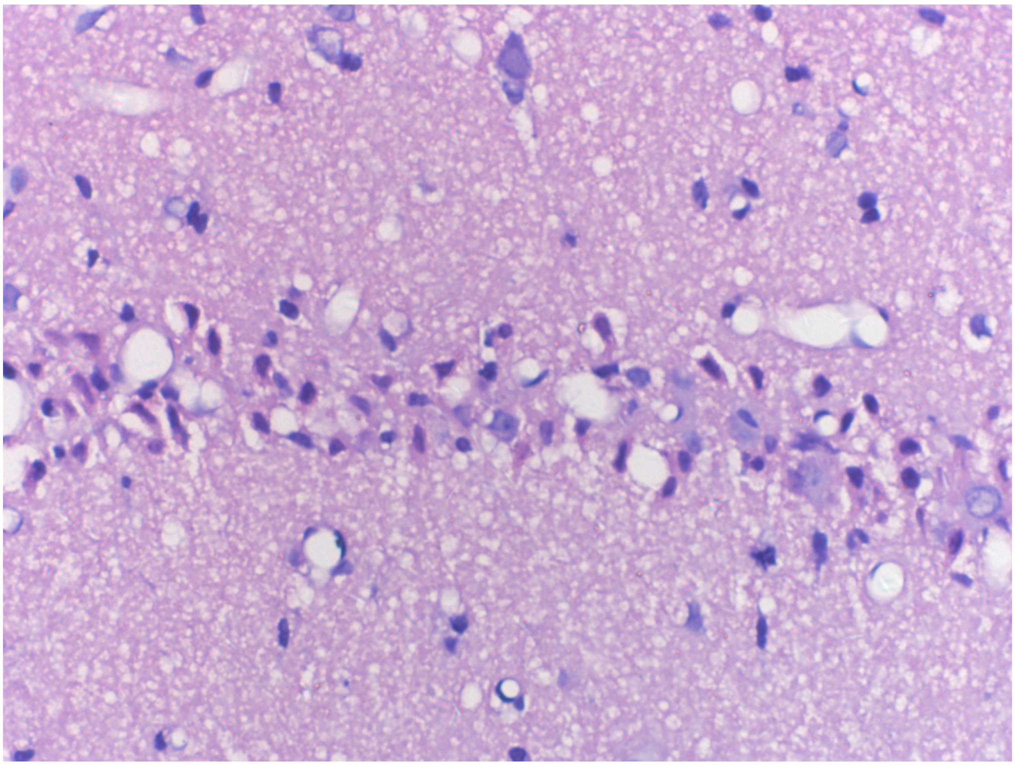

O2_1h_14

(D). ventilated with 100% oxygen for 3 ($O_2$_3h) hour group

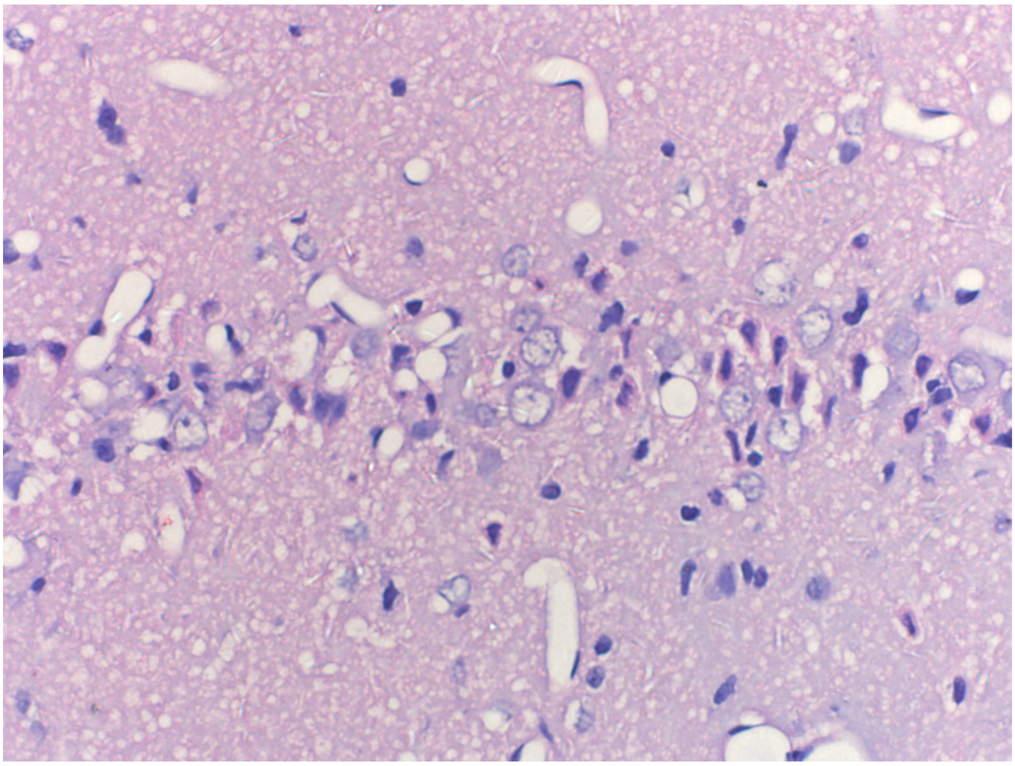

$O_2$_3h_1

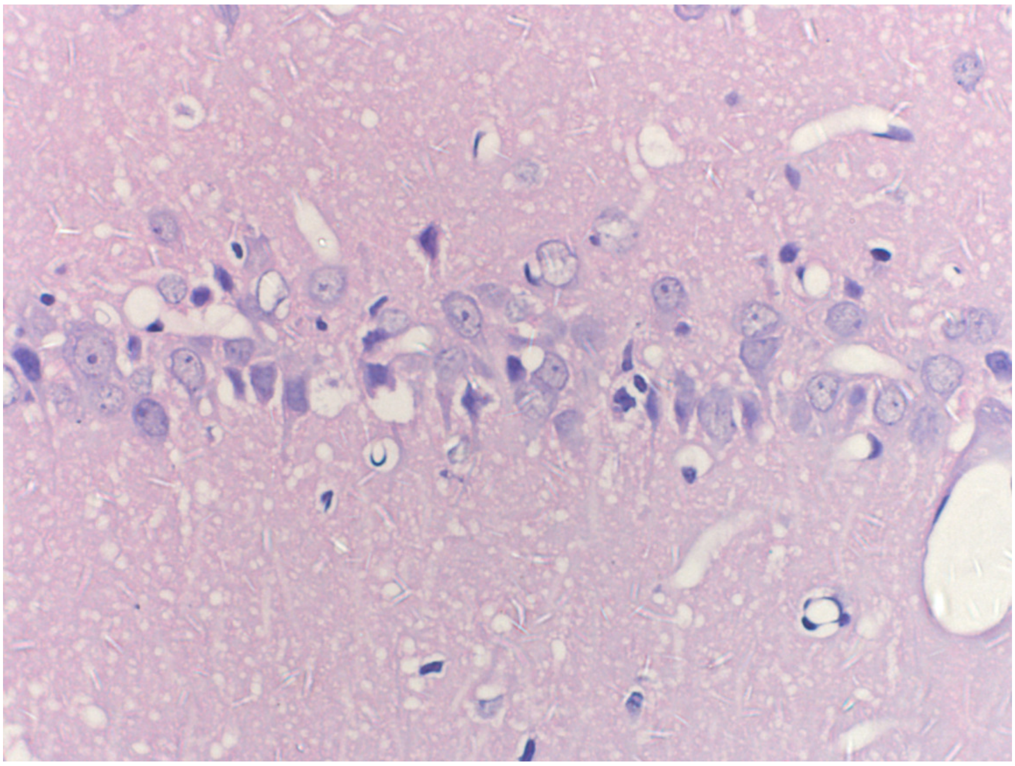

$O_2$_3h_2

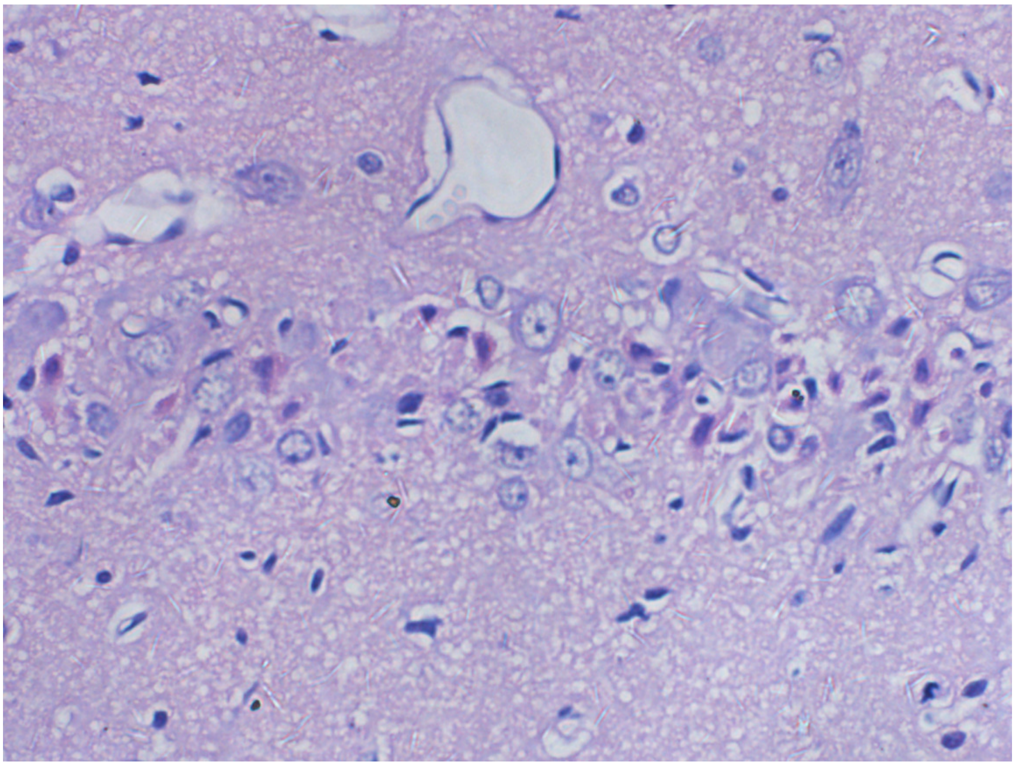

$O_2$_3h_5

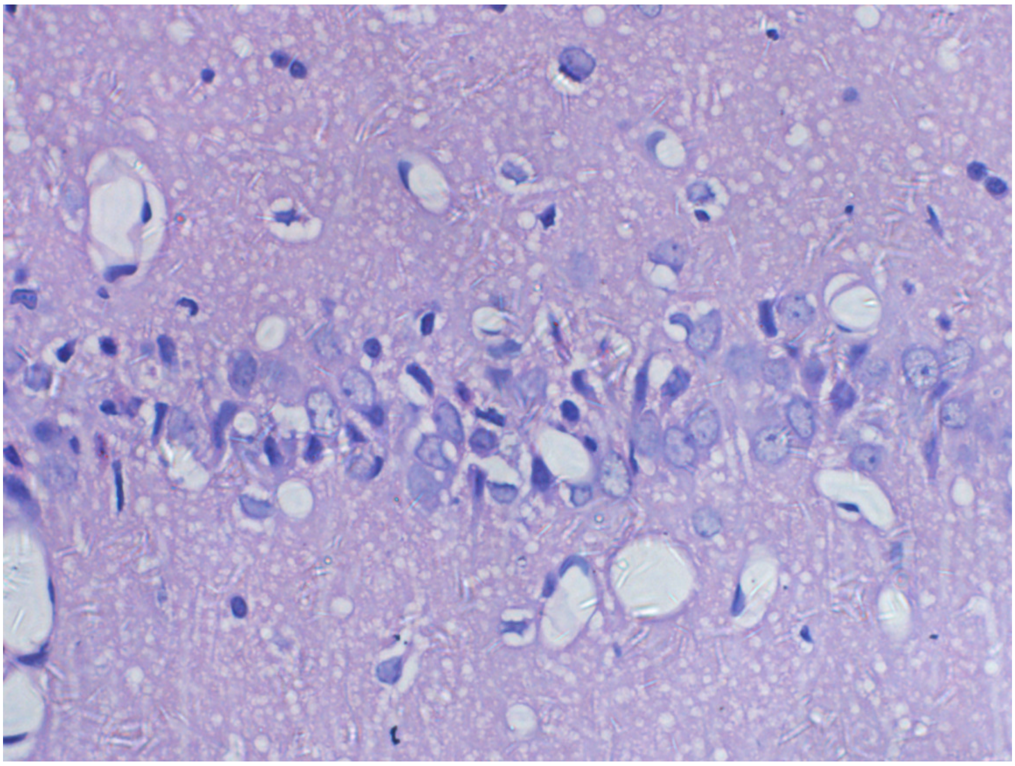

$O_2$_3h_6

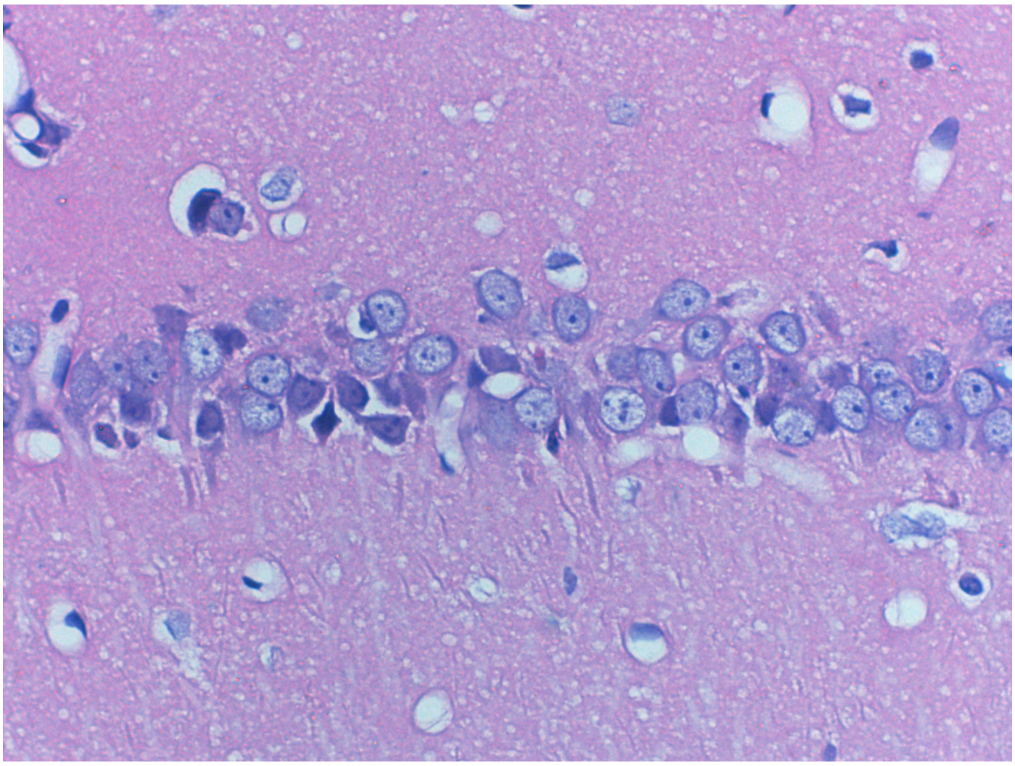

$O_2$_3h_7

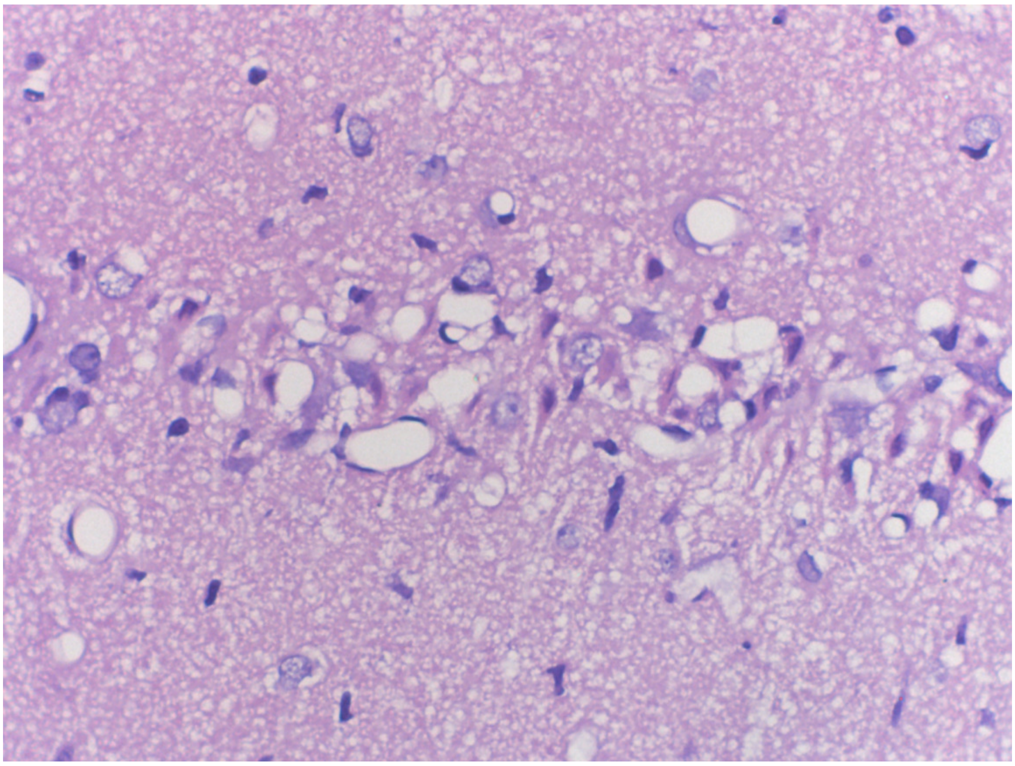

$O_2$_3h_9

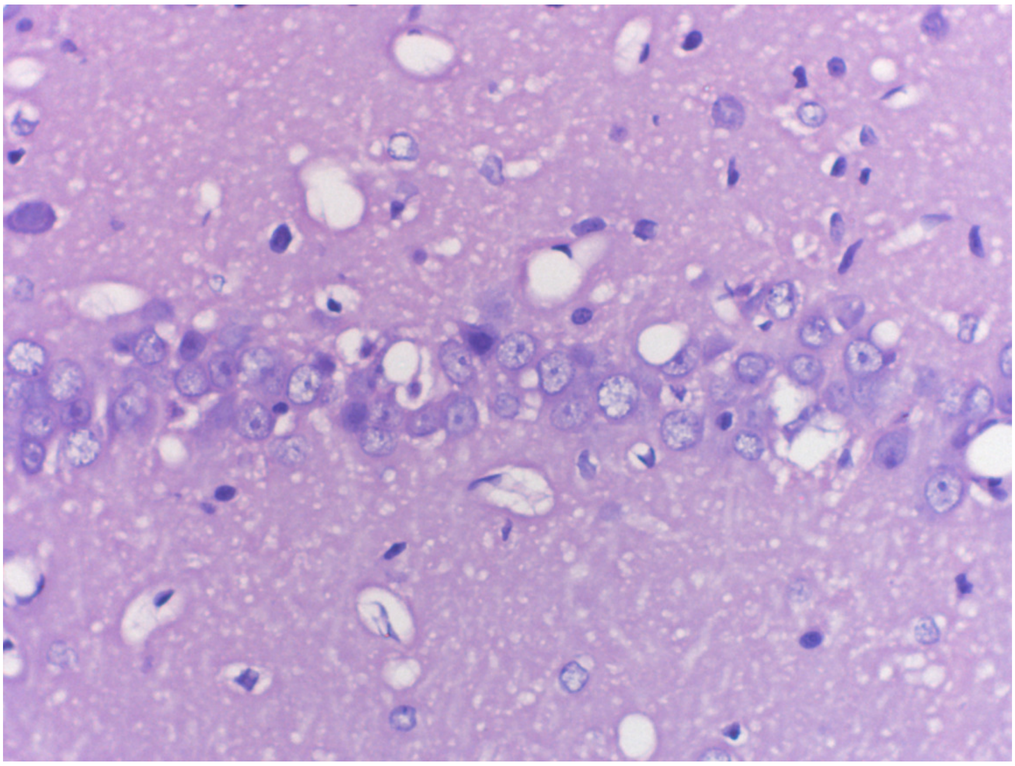

$O_2$_3h_11

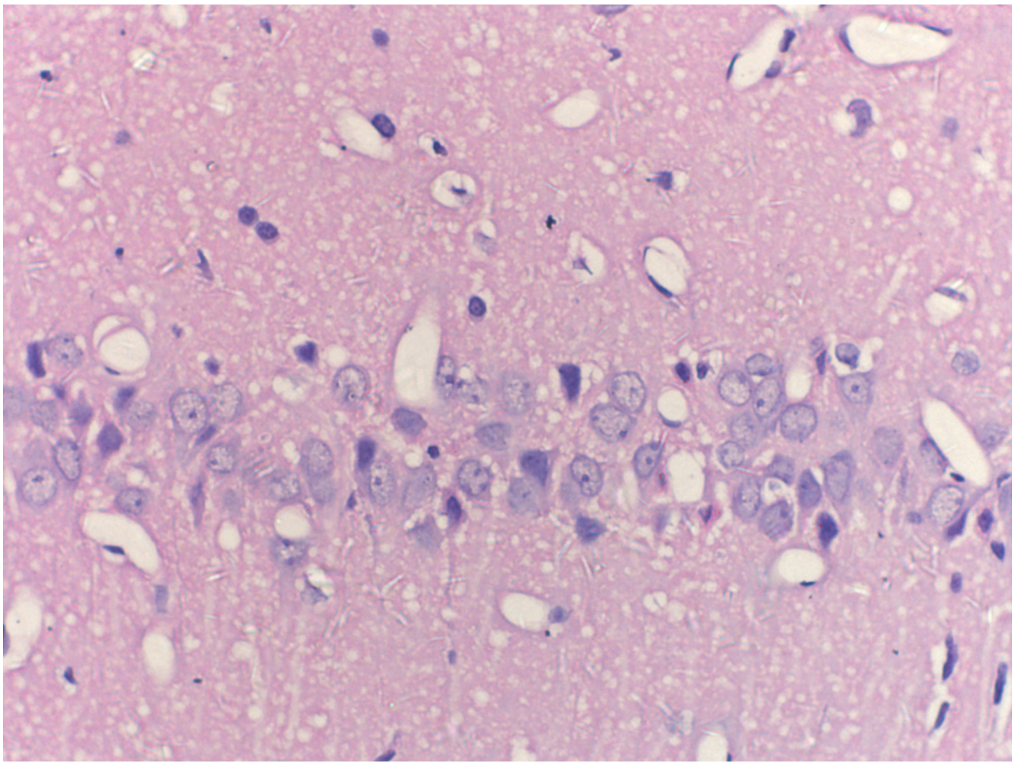

$O_2$_3h_12

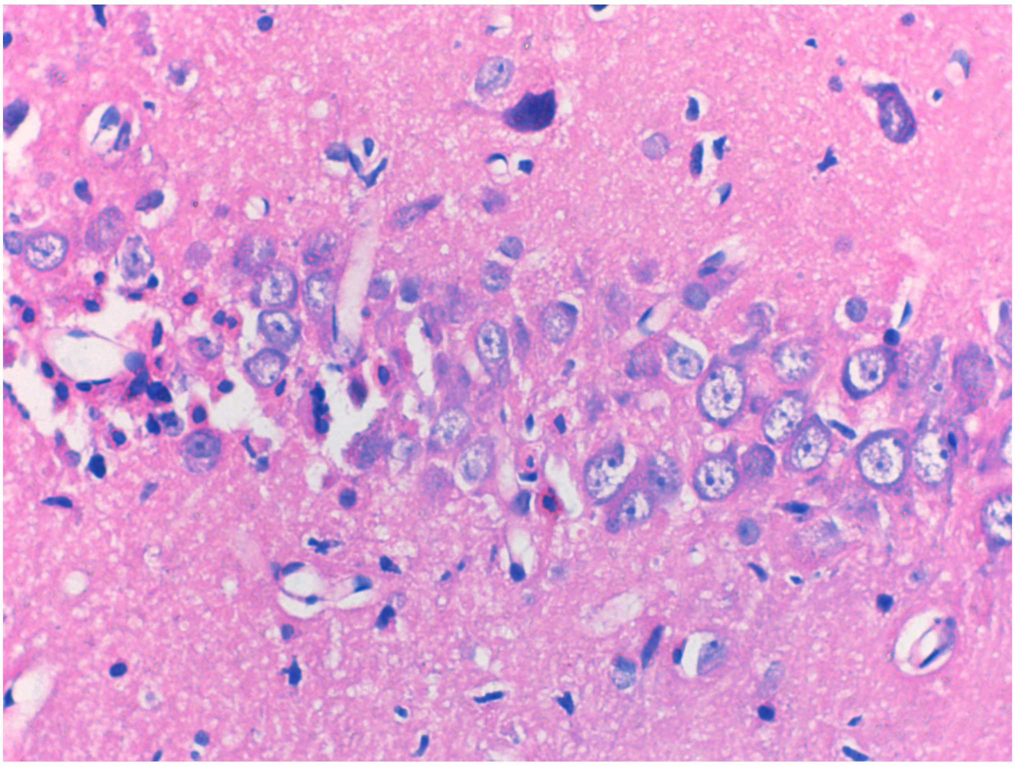

$O_2$_3h_13

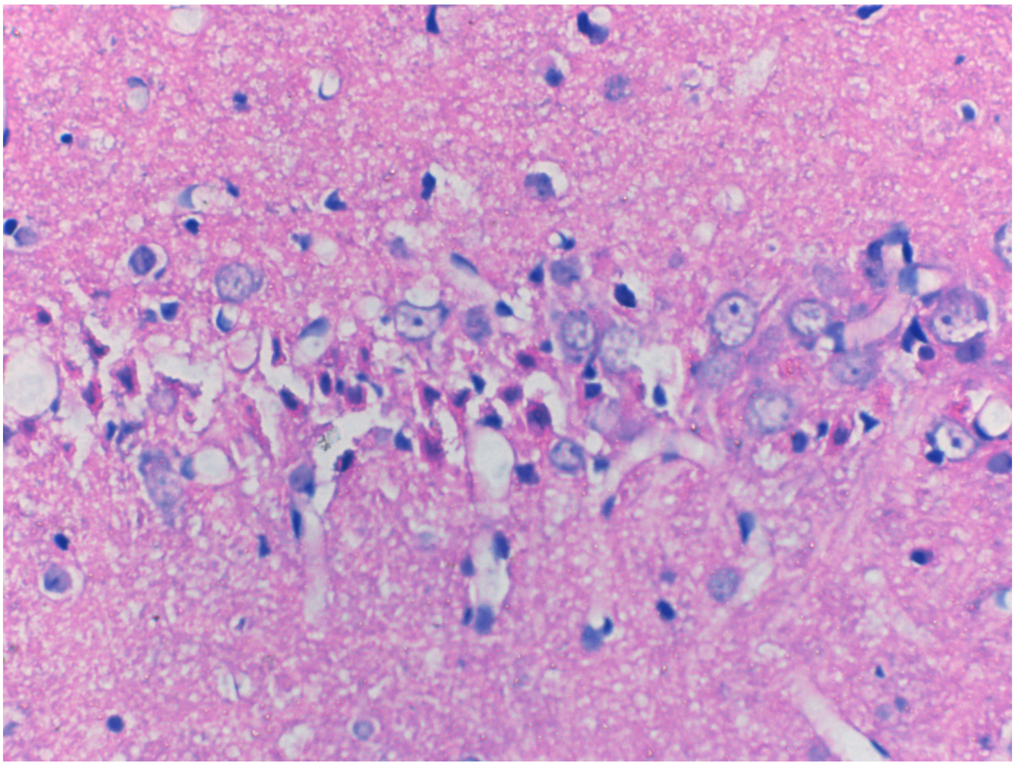

$O_2$_3h_14

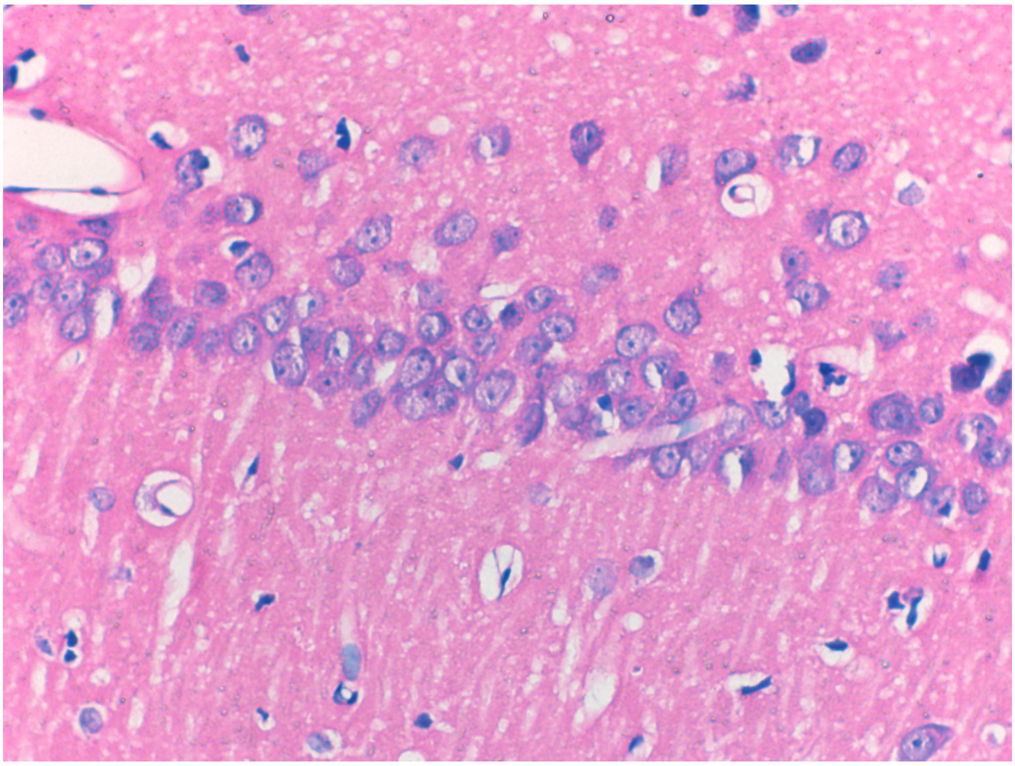

$O_2$_3h_15

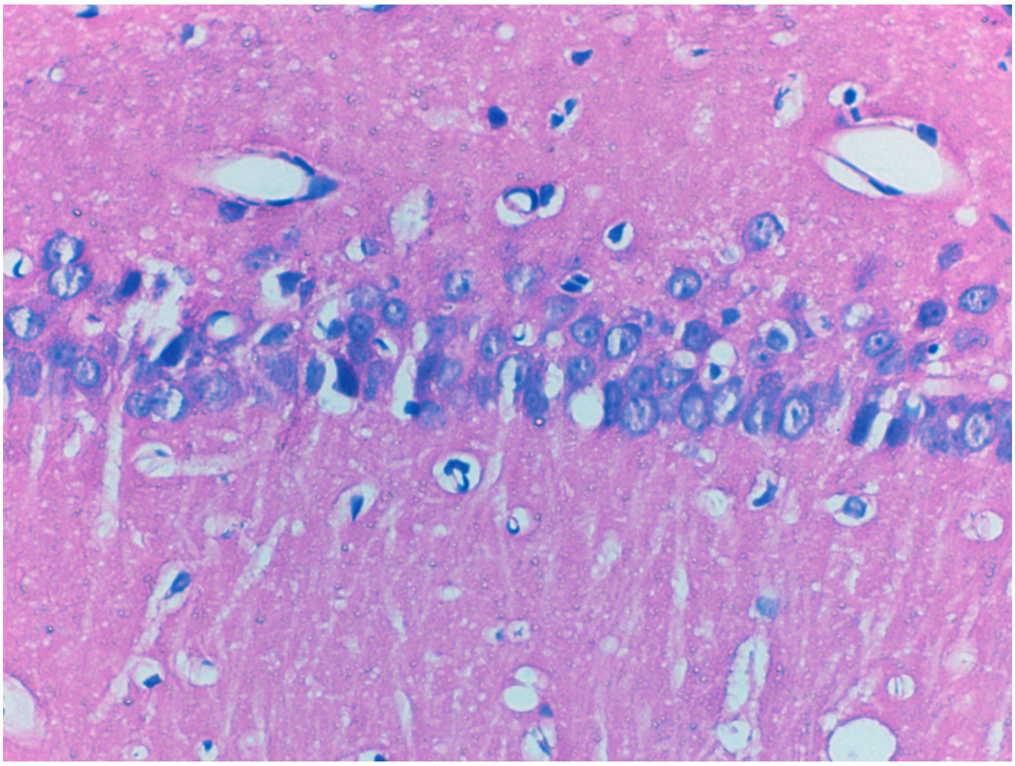

$O_2$_3h_16

(E). ventilated with 100% oxygen for 5 ($O_2$_5h) hour group

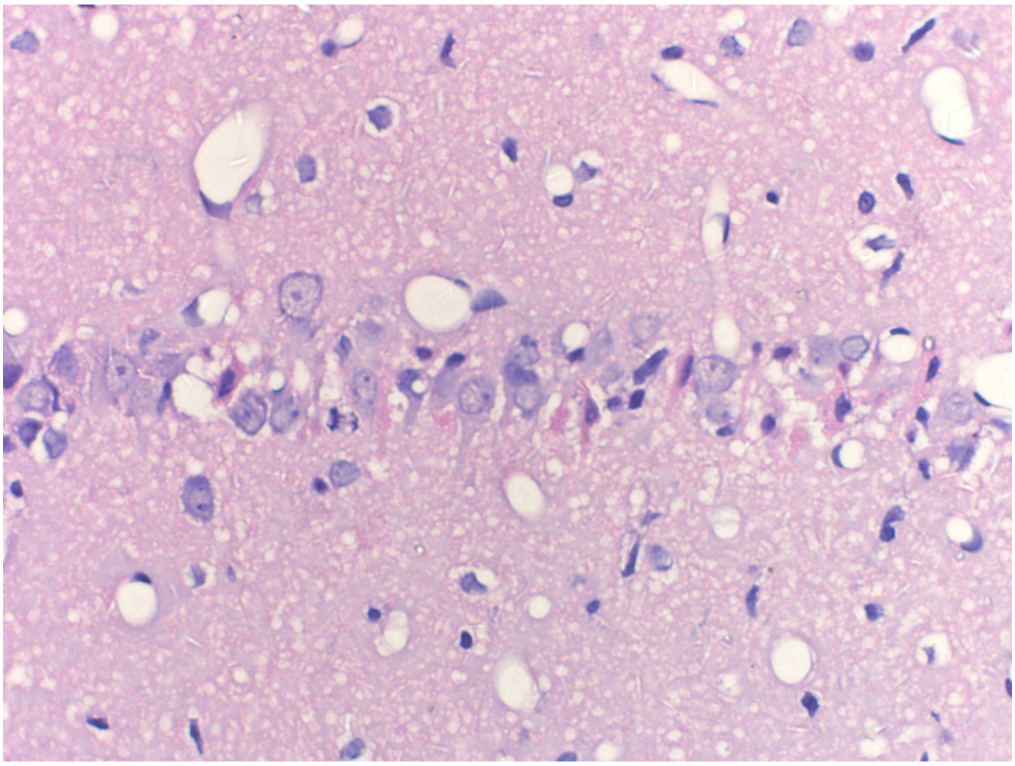

$O_2$_5h_2

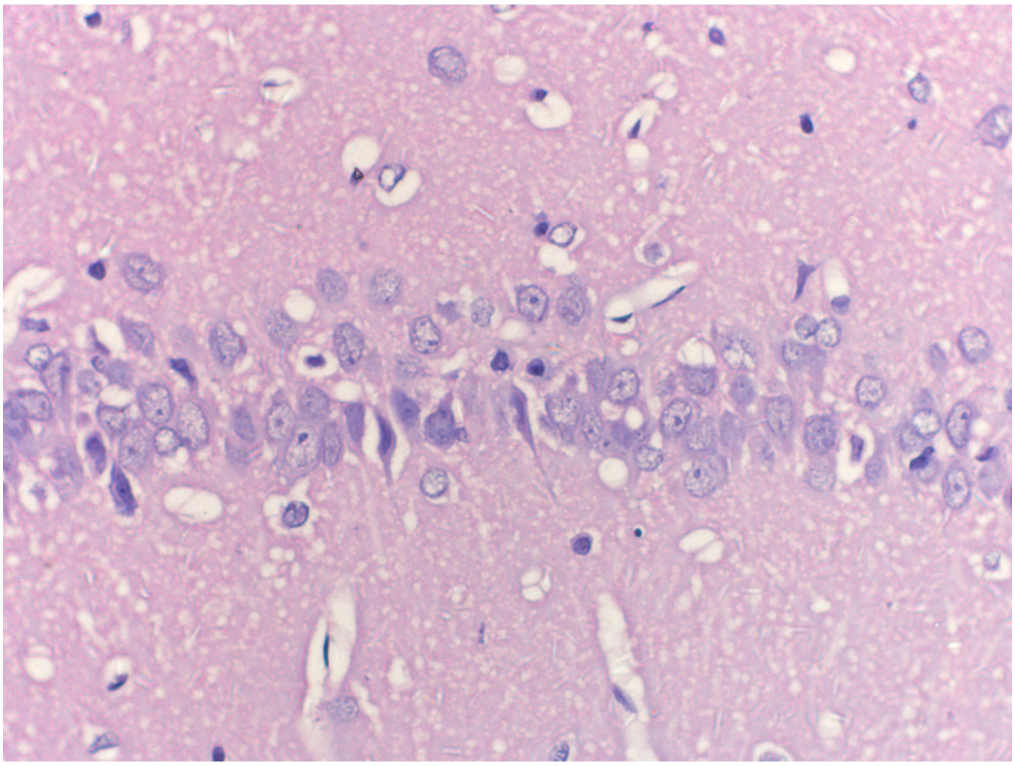

$O_2$_5h_4

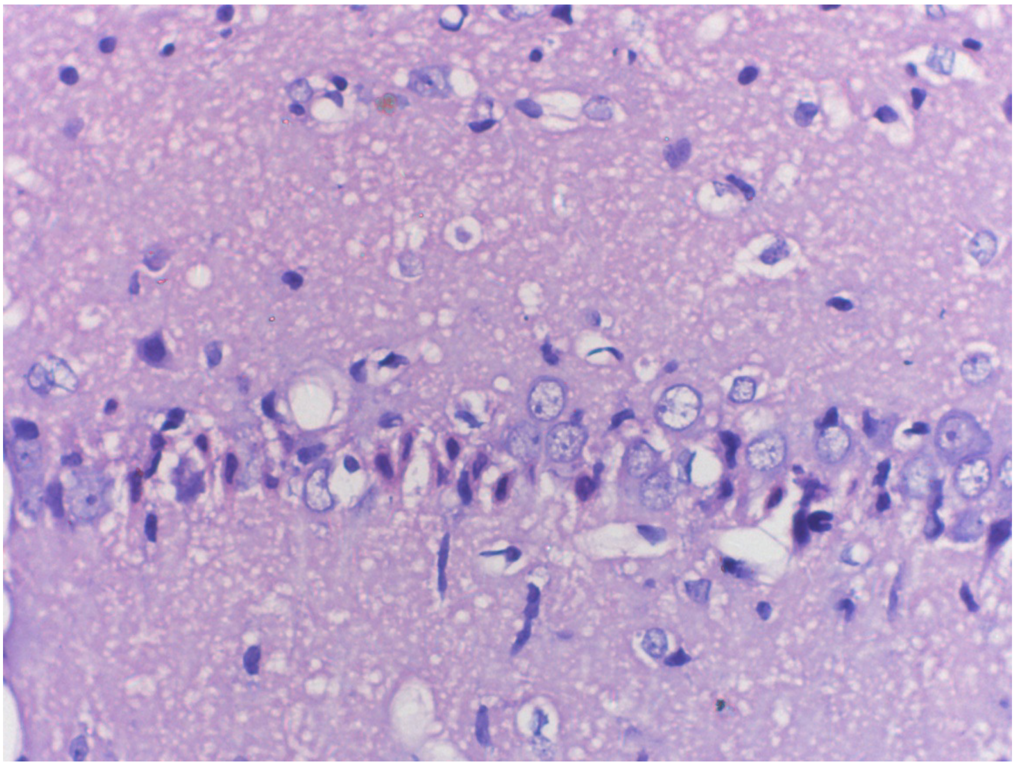

$O_2$_5h_6

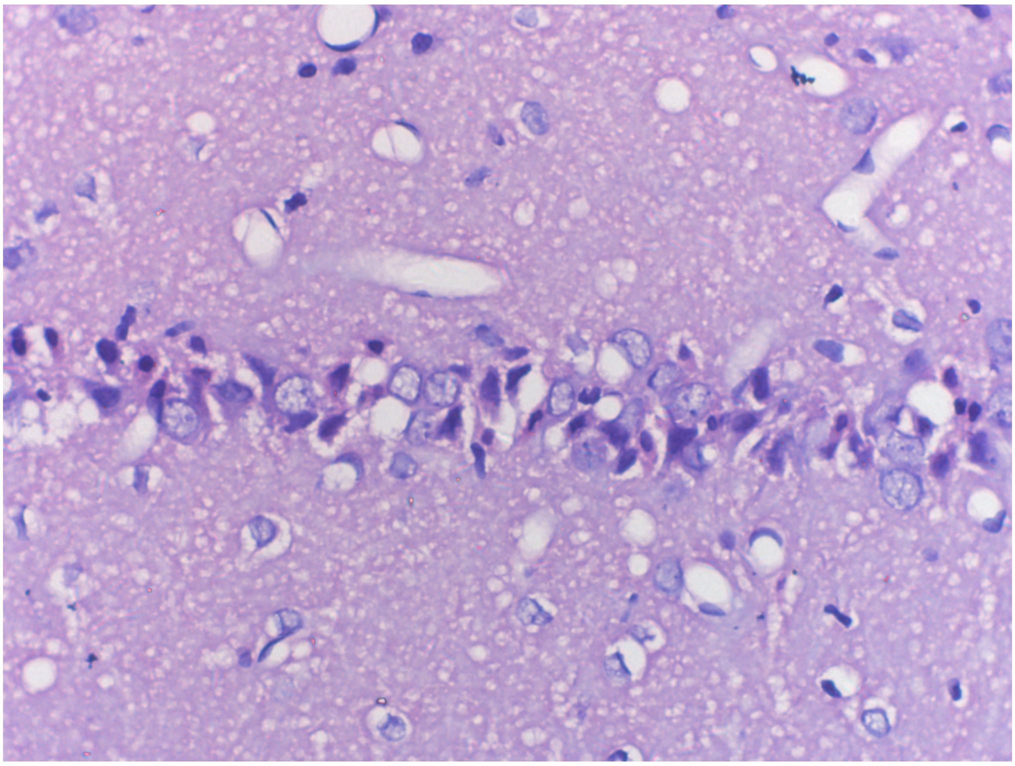

$O_2$_5h_10

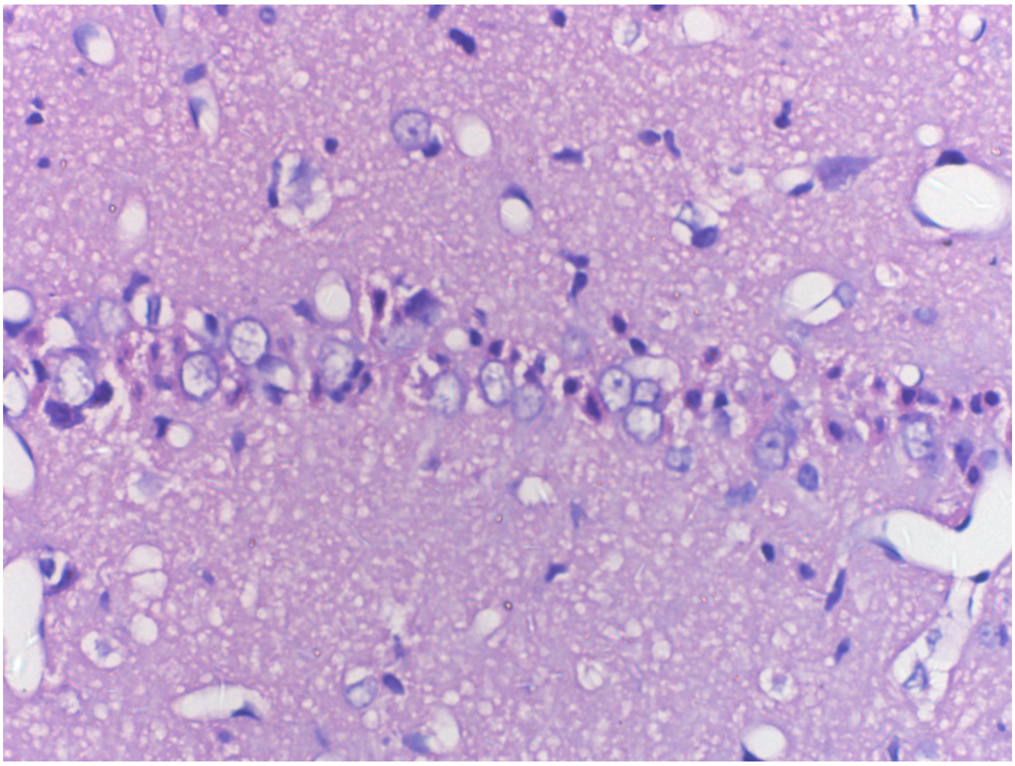

$O_2$_5h_13

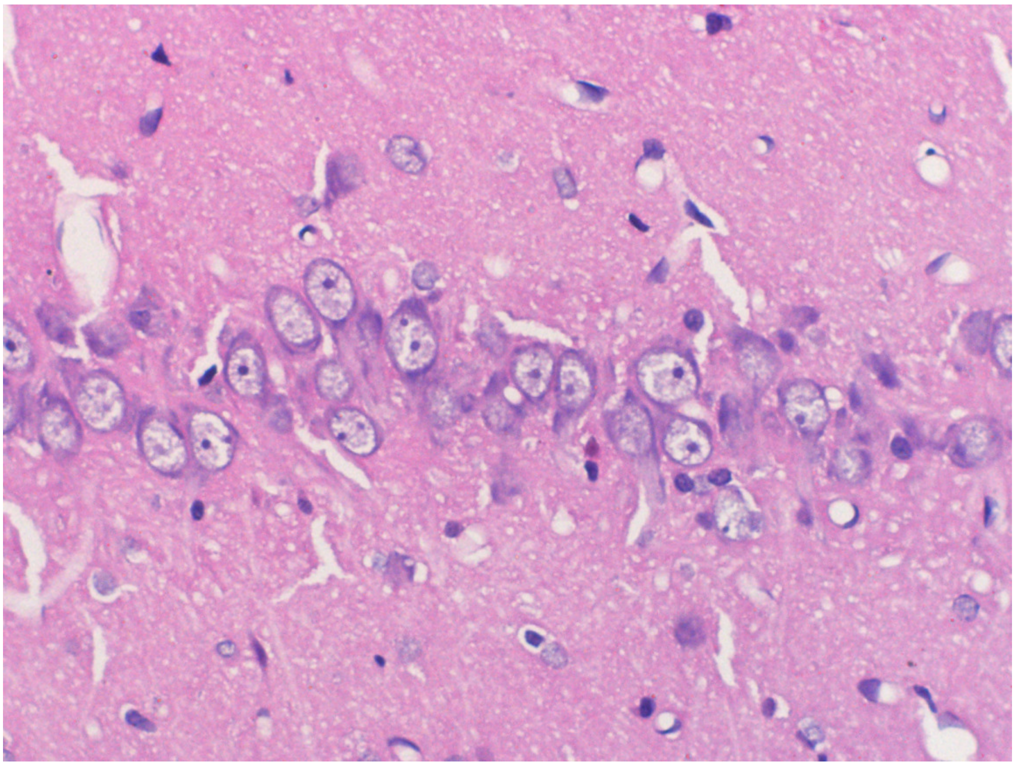

$O_2$_5h_14

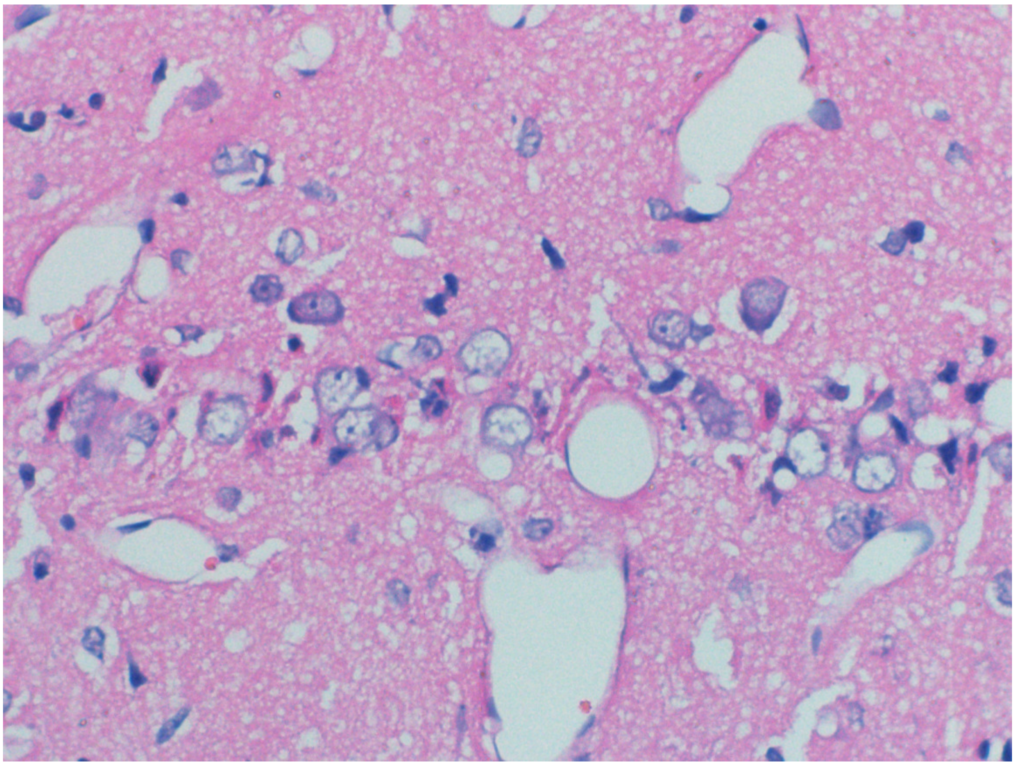

$O_2$_5h_16
